# Suicide risk in Veterans Health Administration patients with mental health diagnoses initiating lithium or valproate: a historical prospective cohort study

**DOI:** 10.1186/s12888-014-0357-x

**Published:** 2014-12-17

**Authors:** Eric G Smith, Karen L Austin, Hyungjin Myra Kim, Donald R Miller, Susan V Eisen, Cindy L Christiansen, Amy M Kilbourne, Brian C Sauer, John F McCarthy, Marcia Valenstein

**Affiliations:** Department of Veterans Affairs, Health Services Research & Development (HSR&D) Center for Healthcare Organization and Implementation Research (CHOIR), MD-152, ENRM VAMC, 200 Springs Road, Bedford, MA 01730 USA; Department of Psychiatry, University of Massachusetts Medical School, Worcester, MA USA; Center for Clinical Management Research, Department of Veterans Affairs, Ann Arbor, MI USA; Serious Mental Illness Treatment Resource and Evaluation Center, Department of Veterans Affairs, Ann Arbor, MI USA; Center for Statistical Consultation and Research, University of Michigan, Ann Arbor, MI USA; Department of Health Policy and Management, Boston University School of Public Health, Boston, MA USA; Quality Enhancement Research Initiative (QUERI), Department of Veterans Affairs, Washington, DC USA; Department of Psychiatry, University of Michigan Medical School, Ann Arbor, USA; VA IDEAS2.0 Center, Department of Veterans Affairs, Salt Lake City, UT USA; Department of Internal Medicine, University of Utah, Salt Lake City, UT USA

**Keywords:** Suicide, Lithium, Valproate, Veterans, Veterans Health Administration, Propensity score, Matching, Discontinuation, Intent-to-treat, Suicidal behavior

## Abstract

**Background:**

Lithium has been reported in some, but not all, studies to be associated with reduced risks of suicide death or suicidal behavior. The objective of this nonrandomized cohort study was to examine whether lithium was associated with reduced risk of suicide death in comparison to the commonly-used alternative treatment, valproate.

**Methods:**

A propensity score-matched cohort study was conducted of Veterans Health Administration patients (n=21,194/treatment) initiating lithium or valproate from 1999-2008.

**Results:**

Matching produced lithium and valproate treatment groups that were highly similar in all 934 propensity score covariates, including indicators of recent suicidal behavior, but recent suicidal ideation was not able to be included. In the few individuals with recently diagnosed suicidal ideation, a significant imbalance existed with suicidal ideation more prevalent at baseline among individuals initiating lithium than valproate (odds ratio (OR) 1.30, 95% CI 1.09, 1.54; p=0.003). No significant differences in suicide death were observed over 0-365 days in A) the primary intent-to-treat analysis (lithium/valproate conditional odds ratio (cOR) 1.22, 95% CI 0.82, 1.81; p=0.32); B) during receipt of initial lithium or valproate treatment (cOR 0.86, 95% CI 0.46, 1.61; p=0.63); or C) after such treatment had been discontinued/modified (OR 1.51, 95% CI 0.91, 2.50; p=0.11). Significantly increased risks of suicide death were observed after the discontinuation/modification of lithium, compared to valproate, treatment over the first 180 days (OR 2.72, 95% CI 1.21, 6.11; p=0.015).

**Conclusions:**

In this somewhat distinct sample (a predominantly male Veteran sample with a broad range of psychiatric diagnoses), no significant differences in associations with suicide death were observed between lithium and valproate treatment over 365 days. The only significant difference was observed over 0-180 days: an increased risk of suicide death, among individuals discontinuing or modifying lithium, compared to valproate, treatment. This difference could reflect risks either related to lithium discontinuation or higher baseline risks among lithium recipients (i.e., confounding) that became more evident when treatment stopped. Our findings therefore support educating patients and providers about possible suicide-related risks of discontinuing lithium even shortly after treatment initiation, and the close monitoring of patients after lithium discontinuation, if feasible. If our findings include residual confounding biasing against lithium, however, as suggested by the differences observed in diagnosed suicidal ideation, then the degree of beneficial reduction in suicide death risk associated with active lithium treatment would be underestimated. Further research is urgently needed, given the lack of interventions against suicide and the uncertainties concerning the degree to which lithium may reduce suicide risk during active treatment, increase risk upon discontinuation, or both.

**Electronic supplementary material:**

The online version of this article (doi:10.1186/s12888-014-0357-x) contains supplementary material, which is available to authorized users.

## Background

Preventing suicide is a global imperative [1], a need for Veterans [2] (especially those with mental health conditions [3]), and a priority for the Veterans Health Administration (VHA) [4]. The psychiatric medication lithium has been previously reported to be associated with uniquely large reductions in risks of suicide death and suicidal behavior [5-7]. Many studies, however, have been nonrandomized, and a 2013 randomized trial meta-analysis did not note any statistically significant differences in suicide deaths between lithium and specific comparison medications [8]. Despite the inclusion of 35 trials, however, just 4 suicide deaths were observed among patients assigned to lithium and 9 suicide deaths among patients assigned to other medications [8]. In contrast, lithium was associated with a significant, and quite substantial, reduction in suicide death compared to placebo (Odds Ratio (OR) 0.13, 95% CI 0.03, 0.66, p = 0.01). A significantly lower risk of suicide death was also noted in a trial of lithium augmentation versus placebo augmentation of antidepressant treatment in major depression, but again was based on extremely few outcomes (3 suicide deaths, all receiving placebo augmentation) [9].

The specific question of whether significant differences in suicide death or suicidal behavior exist between lithium and the anticonvulsant valproate is of particular importance. Valproate is a more commonly-used treatment alternative in the United States than lithium for patients with mood disorders, particularly bipolar disorder. A prominent 2003 nonrandomized study of health maintenance patients with bipolar disorder has only added interest in this comparison by reporting that lithium was associated with statistically significant, > 60% lower risks of suicide death and suicidal behavior compared to valproate [6]. The 2013 randomized trial meta-analysis, however, was unable to evaluate whether differences between lithium and valproate exist in the risk of suicide death, since no suicide deaths were observed in any of the five trials comparing these medications [8]. The meta-analysis did report nonsignificant differences in suicidal behavior between lithium and valproate (OR 0.64, 95% CI 0.30, 1.36, p = 0.24). Valproate is, however, one of many antiepileptic medications that now carries a US Food and Drug Administration-mandated warning stating that antiepileptic medications increase the risks of suicidal thoughts or behavior (for patients taking these medications for any indication). We therefore decided to conduct a nonrandomized cohort study of patients with mental health disorders initiating lithium and valproate using the VHA’s large and detailed clinical databases.

Nonrandomized studies can provide the large samples needed to more easily determine associations between treatments and suicide-related outcomes (especially suicide death). Many previous nonrandomized studies, however, have suffered from substantial methodological limitations, lacking adjustment for many potential confounders, active controls, incident-user designs, uniform follow-up, or intent-to-treat designs [10]. In particular, concerns exist that previous nonrandomized studies may have been confounded through preferential prescription of lithium to patients at low [11] or lower [10] risk of suicide death. It is possible, however, although unestablished, that prescriber behavior has changed over the past 15 years given well-publicized meta-analyses [12,13], treatment guidelines [14], and high-profile studies [6] reporting that lithium treatment may be associated with distinct reductions in suicide death or suicidal behavior.

Using data from this more recent period in which the direction of any confounding between lithium and comparison treatments may have changed, we also sought to reduce the amount of any confounding through methods intended to approximate some of the strengths of randomized trials. In addition to adopting a fixed follow-up time, these methods included: 1) matching patients based on a propensity score [15] of extensive scope and detail [16], and 2) deriving intent-to-treat and post-discontinuation risk estimates [17]. Propensity score matching can permit a greater number of covariates than previously possible to be controlled, creating treatment groups closely similar in prevalence for numerous covariates (similar, in some respects, to a trial). Intent-to-treat estimates ensure that risks arising after treatment discontinuation are considered in judgments of treatment effectiveness. Combining this more recent data with these methodological approaches, we conducted the largest cohort study to date examining whether the risk of suicide death differs between patients initiated on lithium compared to valproate.

## Methods

### Data sources

Demographic, inpatient and outpatient mental and nonmental health treatment records, and outpatient pharmacy prescription data was obtained from the VHA’s National Psychosis and Depression Registries [18] (linked, de-identified healthcare databases of all VHA patients since 1997 with at least one psychotic or depressive disorder diagnosis). This study involved no prospective enrollment of human subjects. Treatment exposure, covariates, and outcome were characterized by information previously recorded during use of the VHA by patients. As with other large healthcare database outcome studies, no informed consent was obtained. This study was approved by the Institutional Review Boards of the Bedford and Ann Arbor Veterans Affairs Medical Centers.

### Study cohort

**Figure 1:**
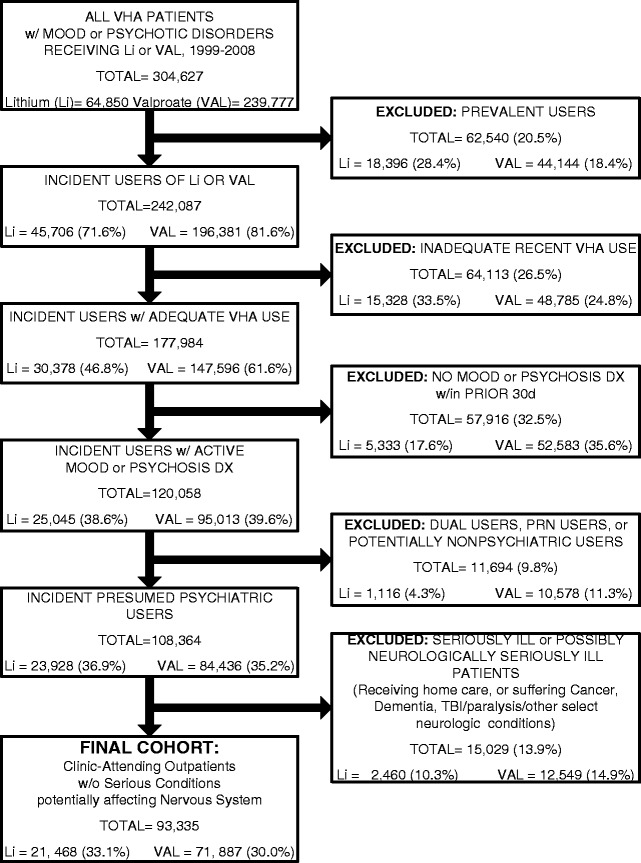
**Flowchart of Study Cohort Derivation.**

Incident users (> 6 months of no lithium or valproate use) with recent VHA utilization (past year and a previous year) were identified among all patients with mood or psychotic disorders within the past 30 days receiving at least one outpatient prescription for lithium or valproate from April 1999 to December 2008. These broad diagnostic inclusion criteria (Additional file 1: Appendix 1) were chosen to maximize statistical power, given the comparatively few suicides expected, even in a large cohort, over a fixed one-year follow-up period, and to facilitate the evaluation of lithium and valproate as broadly useful suicide preventatives. Prior research suggests that any effectiveness of lithium against suicide death is not restricted to patients with bipolar disorder [9,19]. Individuals with potentially nonpsychiatric indications for treatment were excluded (epilepsy, migraine headache, or neuropathy diagnoses in the past 30 days; dementia medication use in the past 180 days; cancer, dementia, skull fracture or traumatic brain injury diagnosis in the past year; traumatic brain injury treatment, home care, or hospice care in the past year; or any nursing home residence or inpatient rehabilitation in the past 2 years). Patients were also excluded if they had started their mood stabilizer on an “as needed” basis (as indicated by receiving an initial prescription designated “prn”) or both mood stabilizers simultaneously (Figure 1).

### Exposure determination

Receipt of lithium or valproate was determined by outpatient prescription fills. The primary analysis examined all individuals initiating either lithium or valproate treatment and followed these individuals until end of follow-up (365 days), suicide death, or death from other causes (i.e., an “intent-to-treat” analysis). As clarification, our study did not examine primary and secondary outcomes per se (the focus consistently being on suicide death), but we did perform primary (intent-to-treat) and secondary analyses of this outcome. Additional secondary analyses examined briefer follow-up times and/or stratified follow-up time by whether individuals were still receiving initial treatment. Individuals were identified as “still receiving initial treatment” if they had not switched to or added the other treatment, nor discontinued the initial treatment (experienced a ≥ 15 day gap between outpatient prescriptions, adjusted for early refills). All other follow-up time was classified as occurring during the period when individuals had “stopped/modified” initial treatment. Since this stopper/modifier group included individuals subsequently resuming either treatment, we also analyzed suicide death risks for individuals over 0–180 days which had stopped initial treatment and not resumed either treatment before suicide, other mortality, or the end of follow-up. Risks observed after treatment discontinuation may reflect risks related to discontinuation of the medication (e.g., “rebound” mania or depression), but may also reflect differences in baseline risk between the treatment groups (i.e., confounding) or differences in selection occurring during follow-up [17].

### Outcome

Date and cause of death (suicide) was obtained from National Death Index files [20] for 1999–2009 using previously established definitions (International Classification of Diseases, Tenth Revision, codes X60–X84, Y87.0, and U03) [21].

### Propensity score modeling

An extensive set of 934 baseline covariates was derived (Table 1 and Additional file 1: Appendix 2) from VHA databases reflecting demographics, diagnoses, general VHA mental and nonmental health healthcare utilization [16], hospitalizations, clinic use, diagnostic testing, current and recent prescriptions (Additional file 1: Appendix 3), diagnosed suicidal behavior, injuries, regional (state-level) suicide risk (Additional file 1: Appendix 2), and prior mood stabilizer treatment. Data in these categories were often modeled over several time periods or with flexible forms (multiple indicator variables). This approach follows the general aims of “high-dimensional” propensity score methodology [16,22], but did not include automated variable generation or selection. Instead, a number of covariates in each domain (Table 1) were generally included, since the full determinants of suicide risk are not well understood, although covariates with a substantial association with treatment exposure (i.e., with which treatment a patient receives) were individually evaluated and removed if they were judged unlikely to be confounders. (Inclusion of variables that are substantially associated with treatment exposure, but which are not confounders, can actually increase confounding from uncontrolled confounders) [23-26].

**Table 1:** **Summary of variables included in the propensity score** ^**a**^

**Type of patient characteristic**	**Covariates included**
**General Covariates**	
Demographics	49 Total Covariates including: Age (5-year categories), Sex, self-reported Race, Ethnicity, Marital Status, Income, Disability Status, Distance to VHA facility, Urban/Rural hospital location, and Year of Medication Start
**Psychiatric Covariates** ^**b**^	
Presenting Diagnosis	9 Variables, including Bipolar I, Bipolar II, Bipolar NOS, Major Depression, Depression NOS, Schizophrenia, Schizoaffective Disorder and Other Psychoses
General Utilization	74 Covariates, including Total VHA Mental Health (MH) Provider Visits x 6 time periods, Total MH hospitalizations x 2 time periods, and Total Diagnostic Interviews, Total Medication Management Visits, Total Individual Psychotherapy Visits, Total Group Psychotherapy Visits (all x 2 time periods), and Total Current MH Medications, Recently Discontinued MH medications, and Possibly Discontinued MH medications
Comorbid Diagnoses	46 Covariates including PTSD, Other Anxiety Disorders, Adjustment Disorders, Personality Disorders, Somatoform Disorders, Impulse Control Disorders, Sleep Disorders, Eating Disorders, Sexual Disorders, Delusional Disorder, ADHD, Development Disorders, Cognitive Disorder NOS, and Dissociative Disorder
Comorbid Substance Abuse Diagnoses	41 Covariates, including 4 Covariates (Abuse, Dependence, Remission from Abuse, and Remission from Dependence for each of the following: Alcohol, Amphetamines, Cocaine, Marijuana, Opioids, Sedatives, Other), with other covariates for Hallucinogen Abuse/Dependence/Remission, Combined Drug Dependence and Remission from Combined Drug Dependence, with and without opioids, Unspecified Dependence, and Alcohol intoxication and Alcohol or Drug psychoses
Suicidal Behavior Diagnoses	9 Covariates, designating if Suicidal Behavior was diagnosed during Nonmental Health hospitalizations, Mental Health hospitalizations, or as an Outpatient, x 3 time periods (0-30 days, 31-180 days, or 181-365 days prior to Lithium or Valproate initiation)
Psychiatric Hospitalizations	10 Covariates, including Covariates designating whether patient was an Inpatient on Start Date, discharged within the last 7 days, 8-30 days, 31-180 days, and 181-365 days, the type of latest hospitalization (Psychiatric, Substance Abuse, Residential/Day program, Domiciliary), and whether any hospitalizations in the last year involved an AMA (Against Medical Advice) discharge
Specific Outpatient Utilization	48 Covariates (all modeled as 0 visits, 1 visits, or 2+ visits): General Mental Health clinic, Psychiatry visits, Psychotherapy visits, Substance Use Disorder visit, Primary Care Mental Health clinic, Health Care for Homeless Veterans, and Substance Abuse and non-Substance Abuse visits, x 2 time periods (0-180 days and 181-365 days)
Current Medications	24 Covariates, including Olanzapine, Risperidone, Quetiapine, Ziprasidone, Aripiprazole, Clozapine, First Generation Antipsychotics, Other Mood Stabilizers, SSRIs, SNRIs, Bupropion, Mirtazapine, TCAs, MAOIs, Benzodiazepines, other Hypnotics, Stimulants, Substance Abuse treatments, and other medications
Recent Medications	24 covariates, designating prescriptions received in last 180 days for same medication categories as “Current Medications,” but for which the patients’ prescribed supply does not extend to the Lithium/Valproate start date
Prior Treatment History	Prior treatment with Any Mood Stabilizer, Prior treatment with Lithium or Valproate
Geographic Suicide Risk	5 variables designating quintiles of Age-Adjusted State Suicide Rates (2000-2007)
**Nonpsychiatric Variables of Possible Particular Relevance to Suicide Risk** ^**b**^	
Nonpsychiatric Diagnoses	7 covariates, including any Acute Injury, any Fracture, Blood Vessel injury, Internal injuries, Open Wounds, Poisoning, Inhalation/Drowning/Asphyxiation injury
Nonpsychiatric Utilization	6 covariates, including Pain Clinic visits (0, 1, 2+) x 2 time periods
Nonpsychiatric Medications	4 variables, including current Opiate Pain Medicine, recent Opiate Pain Medicine, and 2 types of overdose antidotes
**Also Included** ^**b**^ **:**	
Numerous covariates designating Nonpsychiatric Diagnoses, Nonpsychiatric Hospitalizations, Nonpsychiatric VHA Utilization (both the general amount of Nonpsychiatric VHA care received and specific clinics attended and/or care received ), Nonpsychiatric Medications (current and recent), and Nonpsychiatric Diagnostic Tests.	
3 covariates recording prior VHA pharmacy use (any prior use, use in the last 180 days, and use in the last 365 days) were also included to help balance the extensiveness of pharmacy records among recipients.	

^a^Prevalence of each covariate balanced within a standardized difference of < 0.018 in final matched cohort.

^b^All covariates except Demographics relate to care and/or diagnoses made by providers in the VHA health system.

ADHD = Attention Deficit Hyperactivity Disorder; AMA = Against Medical Advice; MAOI = Monoamine Oxidase Inhibitor; MH = Mental Health; NOS = “Not Otherwise Specified”; PTSD = Post-Traumatic Stress Disorder; SNRI = Serotonin-Norepinephrine Reuptake Inhibitor; SSRI = Serotonin-Specific Reuptake Inhibitor; TCA = Tricyclic Antidepressant; VHA = Veterans Health Administration.

### Statistical methods

The propensity score was calculated by logistic regression (c statistic = 0.69). We then 1:1 matched patients initiating lithium and valproate using propensity score calipers of 0.03 (0.2 standard deviations of the propensity score logit) [27,28], achieving 98.7% matching of lithium-initiated patients. Balance in the prevalence of covariates between the treatment groups was assessed using standardized differences (Table 2). Standardized differences are equivalent to Cohen’s *d* effect sizes, with a difference of ≥ 0.10 often considered as indicating significant imbalance [15].

**Table 2:** **Characteristics of Patients Initiating Lithium and Valproate (Propensity-score Matched Sample)**

**Patient characteristic**	**Lithium (n=21194)**		**Valproate (n=21194)**		**Standardized difference**
	**N**	**(%)**	**N**	**(%)**	
**Demographics**					
Age ≥50 years old^a^	10244	48.3	10156	47.9	0.008
*Sex (Female*)^b,c^	2894	13.7	2934	13.8	0.005
*Race, White* ^b^	16748	79.0	16793	79.2	0.005
*Race, Black* ^b^	2825	13.3	2770	13.1	0.008
Married	7416	35.0	7298	34.4	0.012
*State Suicide Rate, 3* ^*rd*^ *quintile* ^*b*^	3305	15.6	3251	15.3	0.007
**Presenting Diagnosis** ^**d**^ (Past 30 days)					
*Bipolar I Disorder* ^b^	9562	45.1	9683	45.7	0.011
*Bipolar Disorder,*	1643	7.8	1661	7.8	0.003
*Not Otherwise Specified (NOS)* ^b^					
*Depressive Disorder,*	4214	19.9	4129	19.5	0.010
*Not Otherwise Specified (NOS)* ^b^					
*Schizophrenia* ^b^	924	4.4	949	4.5	0.006
*Other Psychosis* ^b^	252	1.2	255	1.2	0.001
**Additional Psychiatric Diagnoses** (Past Year)					
*Post-Traumatic Stress Disorder (PTSD)* ^b^	4842	22.8	4749	22.4	0.010
Alcohol Dependence	4426	20.9	4478	21.1	0.006
**Recent Suicidal Behavior Diagnoses** (past 30d, by location where diagnosed)					
Nonmental Health Hospital-Diagnosed	28	0.13	24	0.11	0.005
Mental Health Hospital-Diagnosed	30	0.14	32	0.15	0.002
Outpatient Visit-Diagnosed	144	0.68	147	0.69	0.002
**Recent Suicidal Behavior Diagnoses** (past 31-180d, by location where diagnosed)					
Nonmental Health Hospital-Diagnosed	43	0.20	43	0.20	0.000
Mental Health Hospital-Diagnosed	31	0.15	29	0.14	0.003
Outpatient Visit-Diagnosed	90	0.42	82	0.39	0.006
**Possible Suicidal Behavior-Related Diagnoses** (past year)					
Any Acute Injury	3872	18.3	3884	18.3	0.001
**Recent Discharge from Psychiatric Hospitalization**					
Discharged in past 7 days	2232	10.5	2219	10.5	0.002
Discharged in past 8-30 days	863	4.1	881	4.2	0.004
Discharged in past 31-180 days	2024	9.5	2063	9.7	0.006
**Current Psychiatric Medications**					
*Other Mood Stabilizer(s)* ^b^	2891	13.6	2854	13.5	0.005
SSRI antidepressant	7615	35.9	7666	36.2	0.005
SNRI antidepressant	1988	9.4	2019	9.5	0.005
**Past Treatment History**					
*Prior Mood Stabilizer* ^b^	7503	35.4	7530	35.5	0.003
**Diagnoses, Nonpsychiatric** (past year)					
*Mild Liver Disease* ^b^	1747	8.2	1719	8.1	0.005
**Outpatient Utilization, Nonpsychiatric** (past 180d)					
*Gastroenterology Clinic, 1+ visits* ^b^	1102	5.2	1077	5.1	0.005
**Current Medications, Nonpsychiatric**					
*Thiazide Diuretic* ^b^	1499	7.1	1492	7.0	0.001
*ACE Inhibitor* ^b^	2764	13.0	2736	12.9	0.004
*NSAIDs* ^b^	3491	16.5	3522	16.6	0.004

^a^Age presented in this format (< 50 years vs. ≥ 50 years old) to streamline its presentation within this Table. Age was actually modeled using 11 indicator variables reflecting age groups from < 35 years old, in 5-year intervals, to ≥ 80 years old.

^b^This covariate (designated by *italics* as well as this footnote) had an initial imbalance between the treatment groups of a standardized difference of ≥ 0.10. Please see Additional file 1: Appendix 2, Table S1 if more detail is desired.

^c^The proportion of females in the cohort is low because the Veteran sample is predominantly male.

^d^Percentages for Indicating Diagnoses do not add up to 100%. Some diagnoses were not substantially imbalanced and therefore not included in the Table, although they were included in the propensity score and matched upon (e.g. Major Depression, Bipolar II Disorder, Schizoaffective Disorder, and ≥2 Indicating Diagnoses in past 30 days).

For the analyses of the intent-to-treat cohort and of individuals still receiving initial treatment, we used conditional logistic regression, whereas for individuals stopping or modifying treatment, ordinary logistic regression was used since matching was not rigorously preserved.

Several additional analyses were conducted, including comparing the prevalence between the treatment groups of diagnostically-coded suicidal ideation (V62.84, a code for suicidal ideation first introduced in 2005) in the 30 days prior to treatment initiation among the patients who potentially could receive this diagnosis (the < 50% of the sample initiating treatment in 2005 or later). We also compared the suicide risk associated with the treatment groups prior to matching, conducted a Cox regression analysis, and conducted a sensitivity analysis matching the sample with an alternative propensity score. All analyses were performed using SAS, version 9.3, except the standardized difference calculations (Microsoft Excel 2007).

## Results

A 1:1 propensity-score matched cohort of 42,388 patients (including 102 suicide deaths over 365 days of follow-up) was derived from 93,335 incident users of lithium or valproate (Figure 1). Patients initiating lithium and valproate were generally balanced even prior to matching on a wide variety of psychiatric and nonpsychiatric diagnoses, outpatient and inpatient utilization, and medication covariates: only 17 of 934 covariates (1.8%) exhibited initial standardized differences of ≥ 0.10 between treatment groups. Table 2 demonstrates the close balance achieved after propensity score matching between treatment groups for these 17 initially-imbalanced covariates (i.e., the italicized covariates in Table 2) and 14 other important covariates. Similarly close balance after matching was observed for all other covariates (none of the 934 covariates had a standardized difference of even 0.018 after matching). Despite the general balance in most covariates observed between the treatment groups even prior to matching, propensity score matching led to a substantial reduction in the observed treatment effect estimate (initial 0–365 day Odds Ratio [OR] = 1.45 [lithium/valproate], versus 0–365 day Conditional Odds Ratio [cOR] = 1.22 after propensity score matching) (Additional file 1: Appendix 4).

The treatment groups also displayed very substantial, but highly similar, rates of stopping or modifying initial treatment: 47% of patients initiated on both lithium and valproate were still receiving their initial treatment at 90 days, 24% at 180 days, and only 8% at 365 days (Additional file 1: Appendix Table S1).

Table 3A and 3B provides results for these extensively-matched treatment groups over the first year after medication initiation. No significant difference in suicide death was noted in the primary analysis of all patients initiating lithium versus valproate (intent-to-treat 0–365 day cOR 1.22, 95% Confidence Interval [CI] 0.82, 1.81; p = 0.32). In addition, no significant difference was noted in a secondary analysis among patients during the period within the first year in which they were still receiving initial treatment (cOR 0.86, 95% CI 0.46, 1.61; p = 0.63), nor among patients once they had stopped or modified lithium, compared to valproate, treatment (OR 1.51, 95% CI 0.91, 2.50; p = 0.11).

**Table 3:** **Suicide deaths and rates over time by mood stabilizer treatment**

**A. Primary Analysis (Intent-to-treat, 0–365 days)**								
	All Patients Initiating Treatment (Intent-to-Treat Cohort)							
	Patients Initiating Lithium			Patients Initiating Valproate				
Follow-up Time	Patients, No.	Suicide Deaths, No.	Rate (per 10^6^ person-days)	Patients, No.	Suicide Deaths, No.	Rate (per 10^6^ person-days)	Conditional Odds Ratio (95% CI)	Rate Ratio
0-365 days	21194	56	7.27	21194	46	5.98	1.22^a^ (0.82-1.81)	1.22
**B. Findings Stratified by Initial Treatment Status (0–365 days)**								
	During Exposure to Initial Treatment^b^							
	Patients Initiating Lithium			Patients Initiating Valproate				
Follow-up Time	Patients, No.	Suicide Deaths, No.	Rate (per 10^6^ person-days)	Patients, No.	Suicide Deaths, No.	Rate (per 10^6^ person-days)	Conditional Odds Ratio (95% CI)	Rate Ratio
0-365 days	21194	18	6.71	21194	21	7.68	0.86^c^ (0.46-1.61)	0.87
	During Period After Stopping/Modifying Initial Treatment^d^							
	Patients Initiating Lithium			Patients Initiating Valproate				
Follow-up Time	Patients, No.	Suicide Deaths, No.	Rate (per 10^6^ person-days)	Patients, No.	Suicide Deaths, No.	Rate (per 10^6^ person-days)	Odds Ratio (95% CI)	Rate Ratio
0-365 days	19494	38	7.58	19362	25	5.05	1.51^e^ (0.91-2.50)	1.50

^a^p = 0.32.

^b^The counts of patients “During Exposure to Initial Treatment” include all the propensity score-matched patients, since all patients accrued at least some follow-up time in that status. Counts of suicide deaths among these patients indicate suicide deaths occurring on a day in which the patient was classified as still receiving initial treatment. That is, these counts represent suicide deaths occurring during the period covered by a prescribed supply of medication (without any co-prescription of the other medication), or during the gap(s) permitted after the prescription had ended, up until the day that the first gap of 15 or more days had occurred.

^c^p = 0.63.

^d^The counts of patients “During Period After Stopping/Modifying Initial Treatment” indicates all the patients who reach that status by the end of the follow-up period, since all such patients accrued at least some follow-up time during which they were not still receiving their initially assigned treatment. That is, this is a count of patients modifying their initial treatment by switching to or augmenting with the other medication or discontinuing their initial treatment, either temporarily or permanently. Counts of suicide deaths among these patients among these patients indicate suicide deaths occurring on a day after the patient had exited "During Exposure to Initial Treatment" status, whether by discontinuing or modifying their initial treatment.

^e^p = 0.11.

Secondary analyses of briefer treatment intervals (Table 4A and 4B) indicated an increased intent-to-treat risk of suicide death among all patients initiating lithium over 0–180 days that bordered upon, but did not quite attain, statistically significance (cOR 1.56, 95% CI 0.94, 2.58; p = 0.08), in association with significantly elevated risk of suicide death among patients after stopping or modifying lithium, compared to valproate, treatment (OR 2.72, 95% CI 1.21, 6.11; p = 0.015). This differing risk of suicide death between individuals stopping/modifying lithium, versus stopping/modifying valproate, treatment over 0–180 days was associated almost exclusively with those individuals stopping, rather than modifying, treatment (Table 4, Footnote i: OR 3.61, 95% CI 1.34, 9.73; p = 0.011).

**Table 4:** **Suicide deaths and rates over time by mood stabilizer treatment over briefer time periods (0–90 and 0–180 days)**

**A. Intent-to-Treat Analyses**								
	All Patients Initiating Treatment (Intent-to-Treat Cohort)							
	Patients Initiating Lithium			Patients Initiating Valproate				
Follow-up Time	Patients, No.	Suicide Deaths, No.	Rate (per 10^6^ person-days)	Patients, No.	Suicide Deaths, No.	Rate (per 10^6^ person-days)	Conditional Odds Ratio (95% CI)	Rate Ratio
0-90 days	21194	18	9.35	21194	19	9.87	0.95^a^ (0.50-1.81)	0.95
0-180 days	21194	39	10.20	21194	25	6.54	1.56^b^ (0.94-2.58)	1.56
**B. Findings Stratified by Initial Treatment Status**								
	During Exposure to Initial Treatment^c^							
	Patients Initiating Lithium			Patients Initiating Valproate				
Follow-up Time	Patients, No.	Suicide Deaths, No.	Rate (per 10^6^ person-days)	Patients, No.	Suicide Deaths, No.	Rate (per 10^6^ person-days)	Conditional Odds Ratio (95% CI)	Rate Ratio
0-90 days	21194	15	10.00	21194	17	11.30	0.88^d^ (0.44-1.77)	0.88
0-180 days	21194	17	7.99	21194	17	7.93	1.00^e^ (0.51-1.96)	1.01
	During Period After Stopping/Modifying Initial Treatment^f^							
	Patients Initiating Lithium			Patients Initiating Valproate				
Follow-up Time	Patients, No.	Suicide Deaths, No.	Rate (per 10^6^ person-days)	Patients, No.	Suicide Deaths, No.	Rate (per 10^6^ person-days)	Odds Ratio (95% CI)	Rate Ratio
0-90 days	11227	3	6.98	11185	2	4.75	1.49^g^ (0.25-8.95)	1.47
0-180 days	16138	22	13.00	15958	8	4.78	2.72^h,i^ (1.21-6.11)	2.72

^a^p = 0.87.

^b^p = 0.08.

^c^See Table 3, Footnote b.

^d^p = 0.72.

^e^p > 0.99.

^f^See Table 3, Footnote d.

^g^p = 0.66.

^h^p = 0.015.

^i^Risks observed in patients stopping or modifying initial treatment were almost exclusively observed in patients stopping treatment (rather than modifying treatment or discontinuing and later resuming either treatment):

Patients Stopping Lithium: Suicides = 18; Suicide Rate (per 10^6^ person-days) = 18.3.

Patients Stopping Valproate: Suicides = 5; Suicide Rate (per 10^6^ person-days) = 5.09.

This yields an odds ratio of 3.61 (95% CI 1.34, 9.73) and a rate ratio of 3.60.

**Figure 2:**
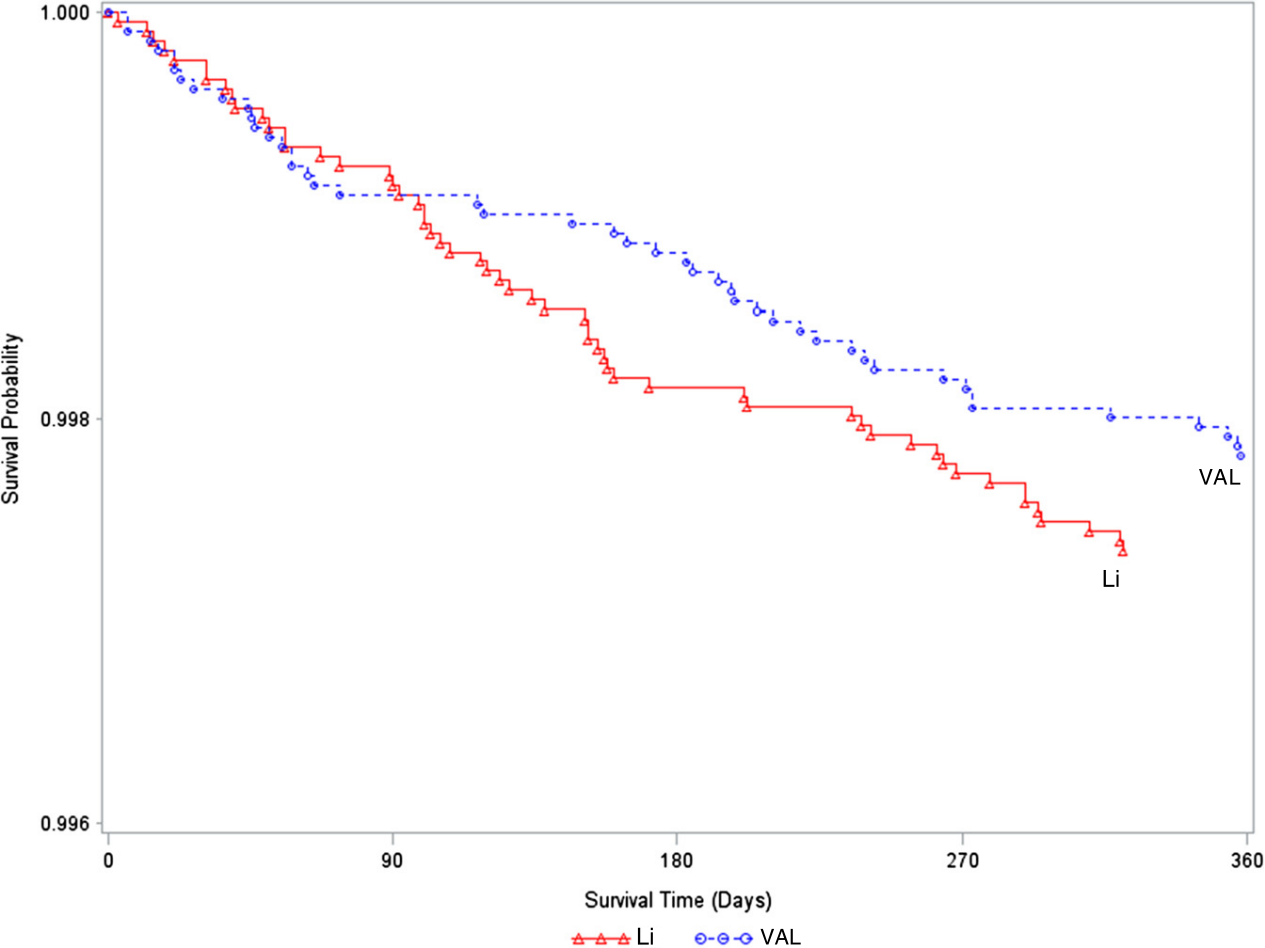
**365-day Survival curve of suicide death by treatment (Intent-to-Treat Analysis).**

Figure 2 presents the intent-to-treat survival curve for suicide death for the lithium and valproate treatment groups. Because of nonproportional hazards (the crossing of the survival curves at approximately 90 days), the interpretation of the survival analysis is less straightforward than, but generally consistent with, the logistic regression results (Additional file 1: Appendix 5).

Table 5 indicates that recent suicidal ideation, when recorded by diagnostic code, was significantly more prevalent among patients initiating lithium than among those initiating valproate (OR 1.30, 95% CI 1.09, 1.54; p = 0.003), for the 19,411 patients for whom these data were available.

**Table 5:** **Presence of V-code (62.84) denoting suicidal ideation in the 30 days prior to lithium or valproate initiation (2005–2008)**

**Patient characteristic**	**Patients initiating lithium**	**Patients initiating valproate**	**Odds ratio (95% CI)**	**P value**
Suicidal Ideation	305	237	1.30 (1.09-1.54)	0.003
No Suicidal Ideation	9478	9391		

## Discussion

This manuscript reports the largest study, to our knowledge, examining lithium’s association with the risk of suicide death. This study is also first to use two design elements (propensity score matching and intent-to-treat analysis) intended to help nonrandomized studies better approximate findings from randomized trials. In this somewhat distinct sample (a predominantly male Veteran sample with a broad range of psychiatric diagnoses), no significant differences in suicide death were observed between lithium and valproate treatment over 0–365 days. This lack of association was observed for both the primary intent-to-treat analyses and for secondary analyses of patients actively receiving their initial treatment (an effectiveness measure traditionally reported in many nonrandomized studies of lithium).

This study’s findings diverge from those of some past meta-analyses [7,29], especially those including nonrandomized studies [7]. Several potential reasons suggest themselves. First, follow-up was only continued for one year (some studies have specifically reported that treatment with lithium for greater than one year was required to observe significant reductions in suicide risk [30-32]). A second reason may relate to characteristics of this sample, including its high rates of treatment discontinuation. High rates of treatment discontinuation would be expected to be especially influential in an intent-to-treat design, since effect estimates would substantially reflect risks observed during periods of nonexposure after discontinuation of treatment. Furthermore, in naturalistic studies high rates of discontinuation can complicate the interpretation of risks even among patients apparently receiving active treatment, since it cannot be clearly ascertained whether or when patients prescribed medication actually consume it. Outcomes for patients who do not start a received prescription or terminate it early may be ascribed to active treatment, but actually relate to nonexposure or treatment discontinuation. Alternatively, some prior studies reporting large associations between lithium treatment and reduced suicide risk may have been biased by inadequately controlled initial confounding or differences in selection occurring during treatment [10,11].

However, two other possibilities deserve consideration, since they are consistent with important elements of our data and would have implications for patients, providers, and healthcare systems. Both relate to the statistically significantly increased suicide risks observed among patients discontinuing lithium at 0–180 days. Risks among patients discontinuing treatment may result from several causes, including: 1) genuine new risks produced by medication discontinuation; and 2) non-treatment-related differences in inherent suicide risk between the treatment groups (i.e., baseline confounding not resolved by the propensity score matching). A third potential cause, differing tendencies between the treatment groups for high-risk patients to discontinue treatment (through self- or provider-based selection), may also contribute to these risks but appears less likely to explain the entirety of our findings (Additional file 1: Appendix 6A). If risks observed after lithium discontinuation relate directly to discontinuing lithium, this finding would appear consistent with a substantial literature documenting pronounced suicide risks upon lithium discontinuation (e.g., suicide rates up to 14-fold greater than during the preceding lithium treatment) [11,12,33]. However, most of these studies lacked control groups. The only prior study comparing suicidal behavior risks among patients discontinuing lithium or discontinuing valproate observed significant increases in suicidal behavior and/or hospitalization risks among patients discontinuing either treatment [34]. In our study, increased risks of suicide death were observed upon discontinuation of lithium, but not valproate.

The substantial increased risks of suicide death observed among patients discontinuing lithium over 0–180 days could also reflect potential confounding remaining after propensity score matching [17,35] (i.e., patients initiating lithium being at higher inherent suicide risk). If active lithium treatment was associated with reduced risk of suicide death, then those reduced risks might largely counterbalance confounding in the intent-to-treat cohort and among those patients still receiving initial treatment. Such confounding might then most clearly manifest as higher suicide risks among lithium-initiating, compared to valproate-initiating patients, after treatment discontinuation.

It is unclear whether much confounding persists in our analysis, given the approximate initial balance observed in numerous measured factors and the further balance achieved after propensity score matching. Several lines of evidence suggest, however, that if any substantial confounding does exist, it likely biases against observing a protective association for lithium. The effect of further increasing covariate balance through propensity score matching was to noticeably reduce initial effect estimates associating lithium with increased suicide death risk. This suggests the initial imbalances in propensity score covariates, although generally small, had the overall effect of biasing associations towards observing higher risks for lithium treatment. Any residual (i.e., remaining) confounding might plausibly bias in the same direction, although this cannot be confirmed with certainty. This bias towards overestimating lithium’s hazards, to the degree it is present, might arise from the patients initiating lithium having more severe mental illness in general, or having a greater prevalence or severity of risk factors for suicide death in particular. Of note, while our data is extensive, it does not include information on several important risk factors for suicide death (e.g., suicidal planning, means, recent stressors, and psychiatric symptoms, and, for some individuals, information on suicidal ideation). Our analysis of diagnostically-coded suicidal ideation found modest but significantly higher rates among patients initiating lithium, even after the propensity score matching. This observation is consistent with the possibility that some degree of remaining confounding exists, biasing towards an overestimate of the risks of suicide death associated with lithium.

A role for chance is also important to consider in interpreting our findings, since a substantial number of comparisons were examined and only three statistically significant associations were observed: among patients discontinuing lithium compared to valproate over 0–180 days (Table 4B), among all patients initiating lithium compared to valproate from 91–180 days, and among patients discontinuing lithium compared to valproate over 91–180 days (Additional file 1: Appendix 5). Nevertheless, while our primary findings over 0–365 days indicate no statistically significant differences between the treatments, results from even a study of this size do not preclude potentially clinically meaningful differences existing between the treatments below the power of this study to detect. Furthermore, the significant risks in patients discontinuing initial lithium treatment over 0–180 days, if not due to chance, generally suggest some degree of nonequivalency between the treatments. These discontinuation-associated risks suggest that lithium is associated with either or both of the following: higher risks of suicide death for some period after discontinuation (in contrast to the discontinuation of valproate), and/or (if some or all of the risks associated with lithium discontinuation reflect baseline confounding) with greater positive benefits against suicide death than suggested by our findings.

### Additional strengths and limitations

Several study limitations should be noted. Data limitations include gaps in VHA prescription records for inpatients and a lack of information about inpatient and outpatient care received outside the VHA, and potential errors in the measurement of covariates. Analytic limitations included the absence of rematching/reweighting of patients during follow-up. This limitation precluded analysis of whether patients experiencing suicidal ideation or behavior during treatment were preferentially discontinued off one of the two treatments, and of whether differences existed during follow-up between the treatment groups in either the initiation of, or persistence with, other psychiatric medications. This study examined typical care, rather than being restricted to monotherapy, unlike some recent studies [34,36]. Given that other psychiatric medications may influence the risks of suicide death [37,38], future studies should consider approaches which reweight patients during follow-up, such as marginal structural models (Additional file 1: Appendices 6A and 8). However, numerous classes of psychiatric medications prescribed at and before lithium or valproate initiation were very closely balanced between the two treatment groups, thus likely producing close similarity in concomitant medications, at least early during follow-up.

In addition, serum medication levels would provide information separate from prescription data about medication persistence, and might enhance future analyses if available. Study findings might have been influenced by the considerable diagnostic heterogeneity of this patient cohort, although each individual diagnosis was closely balanced in prevalence among lithium and valproate recipients and almost 90% of our sample had mood disorder diagnoses (Additional file 1: Appendix 1). Our focus upon suicide mortality (comprehensively documented nationwide, even for patients who leave VHA care) improved outcome ascertainment compared to nonfatal suicidal behavior, but unfortunately limited statistical power. This difference in outcome ascertainment can be substantial: separate studies have estimated that diagnosed nonfatal suicidal behavior may underestimate actual nonfatal suicidal behavior by up to 6-fold to 10-fold [39-41]. This expected underestimate of suicidal behavior has led us to focus our initial investigation, reported here, upon suicide deaths. Similarly, the V-code diagnosis of suicidal ideation we used to assess the potential for residual confounding (Table 5) likely captures only a small fraction of suicidal ideation. However, use of these codes was intended only as an aid to assess the potential presence and direction of residual confounding. For this purpose, diagnoses of suicidal ideation, even if infrequently coded, can provide valuable information, as has been the case in other studies [42].

Propensity score methods may also potentially inadvertently amplify any remaining confounding [23-26]. As pointed out above, such confounding could result from unmeasured differences in mental illness severity or in suicidal risks factors such as suicidal thinking, impulses, or intent. Such amplification is exacerbated if variables are included that are substantially associated with treatment (i.e., lithium or valproate initiation) but not outcome (i.e., suicide death) [25,26]. How much of a bias is typically produced is controversial [25,43]. Nevertheless, the differences between treatment groups in diagnosed suicidal ideation at baseline suggests some unaddressed confounding may remain in this study, and our particular implementation of propensity score matching included extensive variables in some domains (e.g., medical diagnoses) in which only a subset of variables may have been strongly related to suicide risk. (Harris and Barraclough, however, found that 90% of the medical diagnoses they reviewed were significantly associated with suicide death risk [44]). To reduce the potential for amplification of residual confounding, we actively evaluated the plausibility of confounding for those covariates with particularly substantial associations with treatment. Furthermore, a sensitivity analysis targeting this concern by removing a large number of covariates with the weakest apparent associations with suicide death from the propensity score produced only modest effect estimate changes (Additional file 1: Appendix 7). Most importantly, the direction of change in the 0–365 day effect estimates from the unmatched (OR = 1.45) to the matched sample (cOR = 1.22) strongly suggests that overall confounding was most likely reduced by the propensity score matching, not amplified (Additional file 1: Appendix 6B). Therefore, any amplification of residual confounding appears to be sufficiently minor that the propensity score methodology still produced an important reduction in overall confounding. When comparing this study to other studies, however, it is important to note that amplification of residual confounding could have potentially enhanced, to at least a slight degree, an apparent bias in this study against observing a protective association between lithium treatment and suicide death.

### Generalizability, possible concordance with other recent studies, and potential “two-sided” benefit/risk aspects accompanying lithium initiation

Generalizability of this study's findings to non-VHA patients, to patients with the excluded medical conditions (e.g., cancer, head injury, or seizures), to cohorts with differing rates of treatment discontinuation, or to patients that are treated for longer than one year is uncertain. Nevertheless, our findings generally agree with recent studies. With one exception [45], recent nonrandomized studies of suicide or suicidal behavior risk have observed nonsignificant (and typically modest) differences between lithium and valproate specifically, or lithium and anticonvulsants in general [34,36,46-49]. Recently, a small but methodologically-rigorous trial focused on suicidal behavior prevention [50] observed only nonsignificant differences in suicidal behavior between lithium and valproate. Results from this trial (involving 2.5 years of follow-up) and the BALANCE trial [51] (involving 2 years of follow-up) were combined in the recent randomized trial meta-analysis [8], which estimated that lithium treatment was associated with a nonsignificant reduction in nonfatal suicidal behavior compared to valproate (OR 0.64, 95% CI 0.30-1.36, p = 0.24). Thus, the study reported here and the recent trials have both observed nonsignificant intent-to-treat differences between lithium and valproate in suicide death or suicidal behavior. The central estimate for lithium’s effect size, however, did differ in direction between this study (nonsignificantly increased risk of suicide death) and the two trials (nonsignificantly decreased risk of suicidal behavior). This difference might simply be due to chance, residual confounding (possibly augmented by residual confounding amplification), differences in follow-up time (one year versus 2–2.5 years), or differences in outcome (suicide death versus suicidal behavior). However, this difference could also reflect a “two-sided” nature to lithium’s association with the risk of suicide death/suicidal behavior. That is, some degree of decreased suicide/suicidal behavior may be associated with active lithium treatment (thus contributing more greatly to the intent-to-treat estimates from the trials, which had much higher treatment persistence rates), combined with some degree of increased risk associated with lithium discontinuation (which would thus contribute more greatly to the intent-to-treat estimates in our study). A possible “two-sided” association between lithium and the risk of suicide death would be also consistent with the significant differences in baseline suicidal ideation diagnoses between the treatment groups observed in this study (suggesting some residual confounding and thus a greater benefit to active lithium treatment than indicated) and the timing of the emergence of significant risks after lithium discontinuation (suggesting risks associated with discontinuation itself) (Additional file 1: Appendix 6D).

We suspect, although this supposition is inherently somewhat speculative, that the most likely interpretation of our findings includes both phenomena: some genuine association between lithium discontinuation and a short-term increase in the risk of suicide death (compared to valproate discontinuation), along with some residual confounding biasing against lithium. (This residual confounding would imply that active lithium treatment was associated with a larger decrease in suicide risk than estimated in this study). This interpretation would imply that it is difficult to determine whether initiation of lithium in our VHA cohort was associated with a net reduction or increase in suicide risk over the first year of treatment. Our data does suggest, however, that the overall balance of suicide-related risks and benefits from lithium initiation might be increased if greater adherence to initial lithium treatment could be achieved. Research investigating how different health systems could achieve this goal should be a high priority. Possible options include group psychoeducation, which is supported by two clinical trials [52,53]. One of these trials included psychoeducation with other treatment enhancements including detailed illness and treatment histories for each patient, follow-up within 2 weeks of hospital discharge, and a post-discharge “settling in” group [52]. A recent review identified five evidence-based strategies to boost mood stabilizer adherence, including psychoeducational, cognitive-behavioral, interpersonal, and family therapy approaches, as well as systematic care models [54].

### Clinical implications of potential discontinuation-associated risks

Our findings clearly indicate a need for further research of lithium’s association with suicide death or suicidal behavior (Additional file 1: Appendix 8). Until such research resolves the degree to which the increased risks of suicide death observed in patients discontinuing lithium relate specifically to lithium discontinuation (rather than confounding), prudence suggests patient and provider education about the possible risks of treatment discontinuation and close monitoring of patients discontinuing lithium (and potentially valproate [36]) when feasible. Such monitoring is already recommended to limit mood episode recurrences [55]. As discussed above, healthcare systems, providers, and patients should also strive to maximize persistence with lithium treatment once initiated. Finally, when discontinuation does occur, there may be value to gradually discontinuing lithium when appropriate, given that gradual discontinuation has been observed to substantially reduce the risk of mood episode relapse [56,57].

These recommendations concerning the need to minimize lithium and valproate discontinuation, and any risks resulting from this discontinuation, may have relevance well beyond the VHA. The substantial rates of lithium and valproate discontinuation observed here (median time to discontinuation of approximately 90 days) may not be particularly unusual. The observed rates of discontinuation fall within the range provided by the rates of lithium discontinuation reported for a U.S. health maintenance organization (median time to discontinuation of approximately 72 days) [58] and for patients with mood disorders in Denmark being managed primarily by general practitioners, rather than psychiatrists (median time to discontinuation of approximately 181 days) [59].

## Conclusions

In summary, this study did not observe significant benefits for lithium in preventing suicide death compared to valproate among Veterans Health Administration patients over the first year of treatment. This study is notable, however, for high rates of discontinuation of both lithium and valproate, and for the finding of increased suicide risk among patients discontinuing lithium over 0–180 days. If such increased risk largely reflects confounding still persisting in the analysis, such confounding could conceal a clinically meaningful suicide preventative effect for lithium. Alternatively, some or all of the risk among patients discontinuing lithium could represent genuinely greater risks of suicide death related to lithium, compared to valproate, discontinuation. Until further research more fully clarifies the relationships between lithium treatment, discontinuation, and suicide death, patients initiating lithium should be educated concerning the possible risks associated with lithium discontinuation and the need to maximize persistence with lithium treatment, and be monitored closely after discontinuation if feasible. Further research incorporating intent-to-treat approaches is clearly needed, given the possible beneficial or hazardous effect sizes still compatible with this study’s results, the pressing need for interventions against suicide, and the broad potential use of lithium.

## Additional file

Additional file 1:
**Online Supplement for Smith et al., Suicide Risk in Veterans Health Administration Patients with Mental Health Diagnoses Initiating Lithium or Valproate: A Historical Prospective Cohort Study.**

## Abbreviations

CI: Confidence interval
cOR: Conditional odds ratio
OR: Odds ratio
prn: Latin for *pro re nata* (“as the situation demands”), which is used in prescriptions to designate medications that are to be taken only as needed
VHA: Veterans Health Administration
ACE Inhibitor: Angiotensin-converting enzyme inhibitor
AMA: against medical advice
d: Days
D/C: Discharge
Dep: Dependence
DX or Dx: Diagnosis
Dz: Disease
ER: Emergency room
Hosp: Hospitalization
Li: lithium
MH: Mental health
MAOIs: Monoamine oxidase inhibitors
NonMH: Nonmental health
NOS: Not otherwise specified
NSAIDS: Non-steroidal anti-inflammatory medications
PTSD: Post-traumatic stress disorder
SNRI(s): Serotonin-norephinephrine reuptake inhibitor(s)
SSRI(s): Serotonin-specific reuptake inhibitor(s)
TBI: Traumatic brain injury
TCAs: Tricyclic antidepressants
VAL: valproate
w/: with
w/in: within
w/o: without
