# Supplementary material for: Suicide risk in Veterans Health Administration patients with mental health diagnoses initiating lithium or valproate: a historical prospective cohort study
Source: BMC Psychiatry. 2014 Dec 17;14:357. doi: 10.1186/s12888-014-0357-x (PMC4343189; doi:10.1186/s12888-014-0357-x)
Supplement: Additional file 1: — Online Supplement for Smith et al., Suicide Risk in Veterans Health Administration Patients with Mental Health Diagnoses Initiating Lithium or Valproate: A Historical Prospective Cohort Study. [file 12888_2014_357_MOESM1_ESM.pdf]

Online Supplement for Smith et al., Suicide Risk in Veterans Health Administration Patients with Mental Health Diagnoses Initiating Lithium or Valproate: A Historical Prospective Cohort Study

Published in *BMC Psychiatry*

Appendix 1. Diagnostic Codes Included in the Cohort

Appendix 2. Additional Information Concerning Variables Included in the High-Dimensional Propensity Score

Appendix 2 Supplementary Table 1. Key Characteristics of Patients Initiating Lithium and Valproate Both Prior to and After Matching

Appendix 3. Mental Health Medication Covariates Included in the Analysis

Appendix 3 Supplementary Table 1. Mental Health Medications that were Propensity Score- Matched between the Lithium and Valproate Treatment Groups

Appendix 4. Inferences about Residual Confounding Suggested by the Initial Imbalance in the Propensity Score Covariates

Appendix Table 1. Rates of Continuation and Discontinuation of Initial Treatment by Treatment

Appendix 5. Survival Analysis of Suicide Risk by Treatment over 0-90 days, 91-180 days, and 181-365 days

Appendix 4 Supplementary Table 1. Cox Regression Survival Analysis by Time Period since Medication Initiation

Appendix 6. A Potential Integration of Key Study Findings (includes Appendices 6A-6E)

Appendix 7. Modified Propensity Score Analysis

Appendix 8. Suggestions Concerning Future Research

Appendix 1. Diagnostic Codes Included in the Cohort

Since the databases used in this study were initially developed for use in tracking the care delivered to a broad collection of Veterans Health Administration (VHA) patients with depressive or psychotic disorders, a considerable range of diagnostic codes were available for inclusion during database construction. To maximize power and because existing literature suggested than any suicide benefits from lithium might span a variety of diagnoses [1, 2], we decided to retain a broad group of eligible mood and psychotic diagnoses in the cohort. Virtually all cohort members had received a diagnosis of bipolar I, bipolar II, or bipolar NOS, depression NOS, major depressive disorder, schizophrenia, schizoaffective disorder, or "other psychoses" (including Psychosis NOS) within the past 30 days, and the prevalence of each of these diagnostic categories were highly similar between the two matched treatment groups (i.e., within a standardized difference of < 0.018). Table 1 of the manuscript provides the final prevalence by treatment group of those diagnostic categories that exhibited initial substantial imbalances between the treatment groups.

Specifically, patients could enter the cohort with receipt of at least one of a number of ICD-9 codes in the past 30 days prior to lithium or valproate initiation. The most common codes by far were 296.0-296.99 and 311. Much less common were 295.0-295.9, 297.0-297.3, 297.8-297.9, 298.0-298.4, 298.8, 300.4, 301.12, 309.0-309.1, and 293.83. Only a few diagnoses predominated: bipolar disorder, major depression, and depression not otherwise specified; for instance, as Manuscript Table 2 indicates, less than 6% of patients in both treatment groups had a diagnosis of schizophrenia or "other psychoses." In addition, (not shown in Table 2) approximately 5% had schizoaffective disorder. Thus, although our final cohort did include a few individuals with schizophrenia or "other psychoses," the final diagnostic composition consisted of only 11% of individuals with a psychotic disorder, and some of these individuals also had diagnoses of eligible mood disorders within the past 30 days. Furthermore, although our entry criteria did permit some increased diagnostic heterogeneity compared to past studies, as pointed out above, the propensity score-matching did produce an extremely similar prevalence of each individual diagnosis within the two treatment groups.

Appendix 2. Additional Information Concerning Variables Included in the High-Dimensional Propensity Score

DEMOGRAPHICS AND YEAR OF ENTRY

Demographics: Indicator variables were used for age (< 35 years old, ≥ 80 years old, and intervening 5-year age intervals), sex, and race/ethnicity as recorded in Veterans Health Administration databases. (Race information is relevant to studies of suicide risk because suicide rates vary widely based on race. When information on race was missing it was imputed using methods previously developed). In addition, indicator variables were also included for marital status (single/married/separated or divorced/widowed), income, disability status (as indicated by the overall percent of "service connection" for a particular disability or disabilities), distance to Veterans Health Administration (VHA) facility, urban/rural location of the facility where they are obtaining care, and fiscal year of medication start.

UTILIZATION

Utilization variables are derived from VHA clinic stop codes, a set of approximately 500 codes used to categorize each outpatient encounter. These codes result in classifying care provided into considerably broader categories of care than CPT codes used in "high-dimensional" propensity scores [3], reducing the need to consider whether codes should be aggregated or whether information is lost without such aggregation [4].

General Mental Health and Nonmental Health Utilization: We calculated the total number of VHA clinic stop codes relating to encounters with providers over specific time periods. We then used indicator variables to indicate whether, and at what frequency mental health (MH) and nonmental health encounters had occurred over periods as brief as the last 7 days before medication initiation to longer time periods occurring over the previous two years.

For general mental health utilization, we also constructed variables reflecting the total number of hospitalizations (as indexed by discharge dates), and variables dividing total MH provider visits into four subtypes (diagnostic interviews, medical management visits, and individual and group psychotherapy visits) over different time periods. For general nonmental health utilization, we also included variable representing the number of nonmental health hospitalizations and the number of surgery clinic and specialist visits (based on stop codes) during particular time periods. Also, variables were constructed reflecting the total Emergency Room (ER)/Urgent Care visits, lab visits, and presence and absence of a flu shot in the last year (one possible indicator of preventative care).

Finally, for both general mental health and nonmental health utilization, we included indicator variables for the total number of mental health and nonmental health medications, divided into medications that people were receiving on the lithium/valproate start date, the number of medications that they had very recently been taking but for which an active prescription did not exist on the date of lithium/valproate start (termed "Possibly Discontinued"), and the number of medications recently received (within the last 180 days) but not received in the last 30 days ("Recently Discontinued"). The types of medication considered "mental health" is described under the subsection "Medications" below and in Appendix 3. All other medication types were considered "nonmental health medications."

The distinction between "general/basic" utilization and more specific outpatient utilization is somewhat subjective. For instance, we included the total number of lab visits under "general utilization" but included number of X-Rays, EKGs, and other diagnostic tests under "Non-Mental Health Diagnostic Tests."

Mental Health and Nonmental Health Outpatient Utilization: Clinic stop codes were classified with indicator variables to reflect whether a patient had attended no visits of that type, a single isolated visit, or repeated visits (2 or more visits of that type) within a time period. The two time periods examined were the last 180 days prior to lithium/valproate start, and the prior 181 to 365 days before lithium/valproate start. For mental health outpatient utilization, visits were classified as occurring with psychiatrists, psychotherapists, in the general mental health clinic, primary care behavioral health clinic, substance use disorder clinic, or Health Care for Homeless Veterans clinic, with additional indicators for visits involving group treatment.

A much greater variety of stop codes exists for nonmental health outpatient utilization. We chose all stop codes appearing for ≥ 5% of either treatment group in either the last 180 days or days 181 to 365 prior to medication start and other, lower prevalence clinic stop codes thought *a priori* to be of importance as indicating potentially substantially compromised physical health (e.g., pacemaker clinic, etc.).

In addition, nonmental health stop codes also were also used to construct the diagnostic testing module described below.

Mental Health and Nonmental Health Hospitalizations: The VHA uses more than 75 bedsection codes to classify hospitalizations by the type of care received. The 30 bedsections that relate to mental health hospitalizations were classified into 4 larger classes: Psychiatric-focused hospitalizations, Substance Abuse-focused, Residential/Day program, and Domiciliary Program (longer-term housing).

Because suicide risks with relation to mental hospitalization appear to be time-dependent, we focused on capturing timing of hospitalization and the nature of the most recent hospitalization. We constructed multiple indicators to reflect the timing of the latest discharge date relative to medication initiation, as well as characterizing that latest hospitalization into one of the 4 classes of mental health hospitalizations.

With regard to bedsection codes for Nonmental health hospitalizations, a few codes were consolidated when counts were observed to be particularly low (e.g., dermatology bedsection discharges), but in most cases a simple indicator variable was developed to reflect either that the patient's most recent hospitalization had been of that bedsection type, or that any of their hospitalization bedsections in the two years prior to medication start had been of that bedsection type. These latter variables were constructed both as a measure of overall disease burden (of conditions of a severity requiring hospitalization), because for some progressive conditions earlier hospitalizations or diagnoses can actually reflect worse health prognosis [5], and because failing health is one risk factor for suicide. These variables included ICU bedsections, "Step Down" Bedsections, Telemetry Bedsections, General Medicine Bedsections, Specialty Medicine (e.g., Neurology, Cardiology) Bedsections, Surgery Bedsections, etc.

DIAGNOSES

Comorbid Psychiatric and Nonpsychiatric Diagnoses and Indicating Diagnoses: Indicator variables were used to reflect a variety of specific psychiatric diagnoses given in the past year, based on ICD-9-CM. We required all cohort members to have VHA service use in the last year as well as a year prior to the last year, so this time period maximized information about what diagnoses a patient likely actually had. The one exception was diagnoses that served as an indication for treatment (mood or psychotic diagnoses), for which our criteria was more stringent: we required the diagnosis to be entered in the last 30 days. This was done in order to maximize the likelihood that this was the reason the patient was receiving lithium or valproate.

Nonpsychiatric diagnoses are also of importance to address. (In a meta-analysis of literature up through 1993, Harris and Barraclough [6] observed that 19 different nonpsychiatric illnesses were significantly associated with increased suicide risk). Nonpsychiatric diagnoses were aggregated into larger categories based on the comorbid illness categories that make up the Charlson Comorbidity Index and the Elixhauser Comorbidity Index, as per a classification procedure developed for use with administrative databases [7]. For the Charlson index categories, the following 13 (out of the total 17) comorbidity categories were used: Myocardial infarction, Congestive Heart Failure, Peripheral Vascular Disease, Cerebrovascular Disease, Chronic Obstructive Pulmonary Disease, Connective Tissue Disease, Peptic Ulcer, Mild Liver Disease, Moderate or Severe Liver Disease, Diabetes Mellitus without complications, Diabetes Mellitus with complications, Renal Disease, AIDS/HIV Infection.

Elixhauser Comorbidity categories were also included, based on the same reference [7], when these categories were judged not to overlap with the Charlson index categories. The eleven categories included were: Arrhythmias, Weight Loss, Coagulopathies, Pulmonary Circulation Disease, Hypertension without Complications, Hypertension with Complications, Valvular Disease, Neurodegenerative Diseases, Hypothyroidism, Obesity, Anemia from Blood Loss, and Deficiency Anemia.

Multiple indicators were also included to reflect the total number of the Charlson Comorbidity Index conditions, considering all diagnoses received in the past year.

In addition, indicators for other injury-related and a few specific diagnoses that have been linked to suicide risk (progressive, neurodegenerative or autoimmune conditions, and pain diagnosis were

included). Finally, an aggregated smoking indicator was included in this category. Tobacco dependence is recognized as being underdiagnosed in VHA administrative/clinical coding, so we constructed a "recent smoking" variable which assumed a value of "1" if a patient had any of the three in the past year: a diagnosis of Tobacco Dependence, at least one visit to a smoking cessation clinic, or prescription of nicotine replacement therapy or varenicline.

Comorbid Substance Abuse Diagnoses: Seven categories of legal/illicit substance use (alcohol, amphetamine, cocaine, marijuana, opioids, sedatives, other substances) were coded as four different indicators reflecting diagnoses received in the last year: dependence on that particular substance, abuse of that particular substance, remission from dependence of that substance and remission from abuse of that substance. The eighth category, hallucinogens, was coded as only 3 indicators (dependence, abuse, and remission from dependence) because there were insufficient numbers of patients (≤ 5 in one of the treatment groups) diagnosed with remission from hallucinogen abuse in the past year. In addition, indicators were included for combined substance dependence and remission from combined substance dependence, including separate indicators denoting whether this combined dependence included opioids or not. Two indicators were also included for "unspecified" substance dependence. Finally, indicators were included in this category for alcohol intoxication (both a narrow and broad definition) and alcohol or drug psychoses.

Recent Nonfatal Suicidal Behavior Diagnoses: Episodes of nonfatal suicidal behavior, especially those occurring recently, are among the strongest documented risk factors for suicide [8, 9]. However there are concerns that diagnoses may incompletely capture actual episodes of nonfatal suicidal behavior [10]. There are also concerns that outpatient suicidal behavior diagnoses may reflect a history of more remote suicidal behavior rather than behavior necessarily occurring close to the time the diagnoses were entered. To address these concerns a hierarchy was imposed to avoid double-counting of nonfatal suicide behavior episodes between diagnoses recorded during nonmental health hospitalizations, mental health hospitalizations, or during outpatient encounters. Indicator variables were developed reflecting the occurrence of a diagnosis of an episode of nonfatal suicidal behavior over the last 30 days, days 31 to 180, and days 181 to 365 prior to lithium/valproate start. This approach is expected to result in only an approximate indicator of recently diagnosed episodes of suicidal behavior, since a patient could have two separate attempts within a time period that were diagnosed in different settings, and this occurrence would not be reflected in our coding scheme. In addition, the same attempt, if a diagnosis occurred close to the end of a time interval in one setting (e.g., during a non-MH hospitalization), may have been rediagnosed in a second setting in the next time interval. Thus this single behavior episode would appear as two distinct episodes in our coding scheme, not one. Some imprecision of this type is likely unavoidable.

Despite such uncertainties, given the extreme importance of nonfatal suicidal behavior to predicting suicide risk, we felt it was important to incorporate this information when available in our extensive propensity score. Similarly, it was considered important to maintain this distinction concerning the setting of the nonfatal suicidal behavior diagnosis, since an episode diagnosed in a non-mental health hospitalization is likely to be, on average, considerably more serious than diagnoses simply recorded as outpatient diagnoses. It should be recognized that in general diagnoses of nonfatal suicidal behavior are specific but very insensitive [10], although this sensitivity is expected to increase for inpatient diagnoses compared with outpatient (another reason that we made this distinction).

MEDICATIONS

Current and Recent Mental Health Medications: Mental health medication prescriptions active at the time of lithium/valproate start or recently filled (within the last 180 days) were designated into general classes by 24 indicator variables, using a classification system previously developed. This system already uses multiple categories to index antidepressants; for this study we also classified second generation antipsychotics into individual medications (clozapine, olanzapine, risperidone/paliperidone, quetiapine, aripiprazole, ziprasidone). Such an enhanced classification was important given the differential impacts of these medications on both suicide and other mortality risk. An identical number of indicator variables were used to reflect recent but not current prescriptions of medications from these same classes, designating receipt of one or more prescription of that type of medication in the last 180 days in the absence of a prescription whose days' supply includes the start date for lithium/valproate treatment.

For nonmental health medications, a system was developed using medication class code information assigned by the VHA by the VHA national formulary. The VHA assigns every medication administered from the pharmacy into one of more than 1000 classes of medication denoted by the VHA through 5 character "medication class" codes. We took advantage of this classification as a method to logically aggregate prescriptions for related medications (e.g., different thiazide diuretics were able to be aggregated through these codes into a "thiazide diuretic class," different loop diuretics into a "loop diuretic" class, etc.). In many cases, we condensed this "class code" into a 3 character "superclass" code, but in other cases, such as the diuretic example above, in which further distinctions concerning different types of diuretics were judged important, the entire 5 character class code was used. This condensed the approximately 1000 VHA medication classes used by our cohort down to approximately 225 classes/superclasses. Then all revised medication classes present with a prevalence of ≥5% in either treatment group (reflecting number of patients with at least one prescription in the last 180 days, or with a current prescription on the start date of lithium/valproate) were included. In addition, any revised medication classes of less than a 5% prevalence but greater than a 1% prevalence that were judged *a priori* particularly relevant to either suicide or other mortality risk (e.g., warfarin, digoxin, etc.) were included.

Indicators for "Current" medication classes required the patient to have an active prescription with days' supply that included the start date of lithium/valproate, while indicators for "Recent" medication classes required the patient to have had at least one prescription filled in the last 180 days but no active supply at time of lithium/valproate start.

In the rare cases when fewer than 5 individuals had received medications of a particular class currently or recently, this class was either removed from the propensity score model or consolidated with other medication classes. This resulted in small differences, for instance, in the number of classes of current nonmental health medications (54 variables) versus recent medications (55 variables).

Prior Mood Stabilizer Treatment History: We sought to identify incident users of lithium and valproate through the requirement of a ≥ 6 month "clean period" immediately prior to the lithium/valproate initiation date. The "clean period" required that no prescriptions were initiated for either lithium or valproate within this time period, and that no days supply from prior prescriptions of lithium or valproate extend into this period. Nevertheless, some patients had received past treatment in the VHA more remotely that the past 6 months with either mood stabilizers of any type or specifically with lithium or valproate. These patients constituted a clear minority of the treatment groups (≤ 36% of either treatment group had a history of any prior mood stabilizer treatment, and only approximately 12% of either treatment group had a remote prior history of lithium or valproate treatment). To balance our treatment groups in the number of patients with this past medication history, two indicator variables were included in the propensity score, one reflecting past history of treatment with any mood stabilizer, and another indicating a past history of treatment with either lithium or valproate.

OTHER

Nonmental Health Diagnoses Possibly reflecting suicide attempts, Nonmental Health Utilization of special relevance to suicide risk, and Nonmental Health medications of special relevance to suicide risk: Because injuries may occur that are not recognized as representing suicide attempts, we included indicators based on a variety of injury diagnosis codes, reflecting occurrence of these codes in the last year. These indicators included general indicators reflecting any acute injury or any fracture, as well as very specific injuries of concern, such as blood vessel injury, poisoning, and inhalation/drowning/and asphyxiation injury. We also include indicators designating pain clinic use, opiate pain medication use, and designating if patients had received activated charcoal, or naloxone or flumazenil in the past year.

Geographic Suicide Risk: Indicator variables were constructed to classify patients into 5 categories (approximate quintiles) of age-adjusted regional (state-level) suicide risk, based on publically available data from the Centers of Disease Control, which was available from 2000-2007 [11]. Because these statistics would include the suicides of Veterans occurring in this period, there is a theoretical potential for some bias to be introduced by control of this covariate. However, practically, this bias is expected to be exceedingly small, given that > 150,000 suicides occurred across these states over eight years, and our sample accounted for only 102 suicides over that period (< 0.1%). A geographic suicide risk indicator was included because suicide risk has been found to vary substantially from state to state for reasons that are not

completely understood but that might be also expected to influence suicide risk in veterans specifically (e.g., access to firearms).

Nonmental Health Diagnostic Testing: Clinic stop codes reflecting diagnostic procedures over the last 180 days and days 181 to 365 prior to lithium/valproate start were used to construct indicators of the frequency of diagnostic tests over the past year: X-Rays, CT or MRI scans, EKGs, Ultrasound, Echocardiograms, Endoscopy, Pulmonary Function Tests, Nuclear Medicine, and Angiograms (for Angiograms, tests were divided as occurring within the last 180d days and in days 181 to 365 prior to lithium/valproate start).

Three additional variables were included to help balance the extensiveness of pharmacy records among our recipients: any prior use of VA pharmacy, use ≤ 180days prior to LI/VAL start, and use ≤ 365d prior to LI/VAL start.

The Table following this Appendix (Appendix 2 Supplementary Table 1) illustrates how the extensive propensity score-matching strategy balanced the treatment groups on key measured covariates. Because of the much greater number of valproate recipients in our unmatched cohort, the effect of the matching is essentially to select those valproate recipients most similar (in measured covariates) to the lithium recipients. For instance, the single covariate most imbalanced between treatment groups in the unmatched cohort (Bipolar I diagnosis, with a standardized difference of 0.28 between treatment groups) is much more closely balanced in the matched sample, with the two groups having a highly similar prevalence of Bipolar I diagnosis (45.1% versus 45.7% for a standardized difference of 0.011). This prevalence is close to the prevalence of Bipolar I diagnosis in the original, unmatched sample of lithium recipients. In this fashion, the extensive propensity score matching produced a sample, drawing from the original unmatched cohort, that was closely balanced (i.e., all standardized differences after matching < 0.018) on all 934 covariates.*

* Note concerning covariate count: In the manuscript and here, we refer to 934 covariates because these were the number of separate, unique quantities balanced through the extensive propensity score matching. This includes “0 count” indicators for the variables modeled as more than 2 levels (i.e. more than just absent/present). For variables with > 2 levels, but not dichotomous variables, the number of individuals lacking any presence of that indicator (e.g., 0 additional psychiatric medications at baseline) is a separate quantity, rather than simply another form of the information that can be obtained from the count of individuals scoring “1” for the indicator.

Appendix 2 Supplementary Table 1. Key Characteristics of Patients Initiating Lithium (Li) and Valproate (VAL) both Prior to and After Propensity-Score Matching[a]

	UNMATCHED Sample			MATCHED Sample		
**Characteristic**	**Li (n=21468)** n, (%)	**VAL (n=71887)** n, (%)	**Std. Diff.**[b]	**Li (n=21194)** n, (%)	**VAL (n=21194)** n, (%)	**Std. Diff.**[b]
**Demographics**						
Age 50+[c]	10353 (48.2)	36435 (50.7)	0.049	10244 (48.3)	10156 (47.9)	0.008
Sex (Female)[d]	2978 (13.9)	6750 (9.4)	0.140	2894 (13.7)	2934 (13.8)	0.005
Race, White	16994 (79.2)	52493 (73.0)	0.144	16748 (79.0)	16793 (79.2)	0.005
Race, Black	2833 (13.2)	14197 (19.7)	0.177	2825 (13.3)	2770 (13.1)	0.008
Married	7500 (34.9)	26484 (36.8)	0.040	7416 (35.0)	7298 (34.4)	0.012
State Suicide Rate, 3rd quintile	3325 (15.5)	14647 (20.4)	0.128	3305 (15.6)	3251 (15.3)	0.007
**Indicating Diagnosis**[e] (Past 30 days)						
Bipolar I	9737 (45.4)	22811 (31.7)	0.283	9562 (45.1)	9683 (45.7)	0.011
Bipolar NOS	1686 (7.9)	3630 (5.0)	0.114	1643 (7.8)	1661 (7.8)	0.003
Depression NOS	4233 (19.7)	21693 (30.2)	0.243	4214 (19.9)	4129 (19.5)	0.010
Schizophrenia	924 (4.3)	6605 (9.2)	0.196	924 (4.4)	949 (4.5)	0.006
Other Psychosis	252 (1.2)	1914 (2.7)	0.109	252 (1.2)	255 (1.2)	0.001
**Additional Psychiatric Diagnoses** (Past Year)						
PTSD	4894 (22.8)	20011 (27.8)	0.116	4842 (22.8)	4749 (22.4)	0.010
Alcohol Dep	4499 (21.0)	15713 (21.9)	0.022	4426 (20.9)	4478 (21.1)	0.006
**Suicidal Behavior Diagnoses** (Suicide Attempt) (past 30d, by location where diagnosed (Dx)						
NonMH Hosp Dx	28 (0.13)	122 (0.17)	0.010	28 (0.13)	24 (0.11)	0.005
MH Hosp Dx	30 (0.14)	129 (0.18)	0.010	30 (0.14)	32 (0.15)	0.002
Outpatient Dx	145 (0.68)	507 (0.71)	0.004	144 (0.68)	147 (0.69)	0.002
**Suicidal Behavior Diagnoses** (Suicide Attempt) (past 31-180d)						
NonMH Hosp Dx	44 (0.20)	89 (0.12)	0.020	43 (0.20)	43 (0.20)	0.000
MH Hosp Dx	32 (0.15)	87 (0.12)	0.008	31 (0.15)	29 (0.14)	0.003
Outpatient Dx	91 (0.42)	276 (0.38)	0.006	90 (0.42)	82 (0.39)	0.006
**Possible Suicidal Behavior-Related Diagnoses** (past year)						
Any Acute Injury	3950 (18.4)	13569 (18.9)	0.012	3872 (18.3)	3884 (18.3)	0.001
**Psychiatric Hospitalizations**						
D/C past 7 days	2260 (10.5)	9821 (13.7)	0.096	2232 (10.5)	2219 (10.5)	0.002
D/C past 8-30d	879 (4.1)	3469 (4.8)	0.035	863 (4.1)	881 (4.2)	0.004
D/C Past 31-180d	2062 (9.6)	7293(10.1)	0.018	2024 (9.5)	2063 (9.7)	0.006

Appendix 2 Supplementary Table 1. (continued)

**Current Psychiatric Medications**						
Other Mood Stabilizer(s)	3009 (14.0)	6875 (9.6)	0.138	2891 (13.6)	2854 (13.5)	0.005
SSRI antidep	7700 (35.9)	28496 (39.6)	0.078	7615 (35.9)	7666 (36.2)	0.005
SNRI antidep	2046 (9.5)	4993 (6.9)	0.094	1988 (9.4)	2019 (9.5)	0.005
**Past Treatment History**						
Prior Mood Stabilizer	7680 (35.8)	20795 (28.9)	0.147	7503 (35.4)	7530 (35.5)	0.003
**Diagnoses, Nonpsychiatric** (past year)						
Mild Liver Dz	1892 (8.8)	3308 (4.6)	0.169	1747 (8.2)	1719 (8.1)	0.005
**Outpatient Utilization, Nonpsychiatric** (past 180d)						
Gastroenterology Clinic, 1+ visits	1197 (5.6)	2466 (3.4)	0.104	1102 (5.2)	1077 (5.1)	0.005
**Current Medications, Nonpsychiatric**						
Thiazide Diuretic	1515 (7.1)	7650 (10.6)	0.126	1499 (7.1)	1492 (7.0)	0.001
ACE Inhibitor	2784 (13.0)	12320 (17.1)	0.117	2764 (13.0)	2736 (12.9)	0.004
NSAIDs	3516 (16.4)	14738 (20.5)	0.106	3491(16.5)	3522 (16.6)	0.004

[a] A partial version of this Table appears as Manuscript Table 2. Since the degree of imbalance in these variables occurring prior to matching may be of interest to some readers, we present this Table again with 5 extra columns to report the prevalence of these covariates in the sample prior to matching, and to show the reduction in imbalance resulting after the extensive propensity score matching.

[b] Std. Diff = Standardized Difference.

[c] Age presented in this format (< 50 years old vs. ≥ 50 years old) to streamline its presentation within this Table: age was actually modeled using 11 indicators reflecting age groups from < 35 years old in 5-year intervals to ≥ 80 years old.

[d] The proportion of females in the cohort is low because the veteran sample is predominantly male.

[e] Percentages for Indicating Diagnoses do not add up to 100% because some diagnoses are not substantially imbalanced and therefore not listed in this Table (e.g., Major Depression, Bipolar II Disorder, ≥2 Indicating Diagnoses in past 30 days), although they were included in the propensity score and balanced through matching.

ABBREVIATIONS: Dep = Dependence; D/C =Discharge, NonMH Hosp Dx = Diagnosed during a Non-Mental Health hospital stay, MH Hosp Dx = Diagnosed during a Non-Mental Health hospital stay, Outpatient Dx = Diagnosed during an outpatient visit, SSRI = Serotonin-Specific Reuptake Inhibitor, antidep = antidepressant, SNRI = Serotonin-Norepinephrine Reuptake Inhibitor, Dz = Disease.

Appendix 3. Mental Health Medication Covariates Included in the Analysis

Because psychiatric medications are of particular importance in both helping to index the severity of various psychiatric diagnoses and also as potential direct influences on suicidal behavior (e.g., clozapine), we sought to control for a wide variety of psychiatric medications that cohort members might be receiving. We also sought to produce, through propensity score-matching, two cohorts that were not only similar in the psychiatric medications that patients were currently receiving, but also medications that they have recently been receiving (within the last 6 months) but were not receiving currently. Such medications may have been treatments that they or their provider deliberately decided to stop, or intended to continue but were not successful in so doing, or for which they were experiencing only a brief interruption in treatment that happened to occur in proximity to their lithium/valproate treatment initiation date. An additional reason it is important to control for concomitant medications is that a current or recent history of receiving a psychiatric medication may also influence the subsequent psychiatric medications a patient might receive.

Table 1 of the manuscript (and Appendix 2 Supplementary Table 1) lists only those medication classes (i.e., other mood stabilizers, SSRI antidepressants, SNRIs) for which a substantial initial imbalance occurred between the treatment groups. Because of the importance of these covariates, Appendix 3 Supplementary Table 1 below lists all the psychiatric medications or medication classes that were controlled in our analysis. Each of these categories was balanced between the treatment groups to a standardized difference of < 0.018 for each of the time periods.

Appendix 3 Supplementary Table 1. Listing of Mental Health Medications that were Propensity Score-Matched between the Lithium and Valproate Treatment Groups
(24 medication/medication classes x 2 time periods)

**CURRENT MENTAL HEALTH MEDICATIONS** *(24 variables)*<br>*(active prescription on Li / VAL start date)*	
Other Mood Stabilizers<br>(carbamazepine, lamotrigine, etc.)	TCAs
	MAOIs
Olanzapine	Benzodiazepines
Quetiapine	Other Hypnotics
Risperidone	Buspirone
Ziprasidone	Stimulants
Aripiprazole	Disulfarim (Antabuse)/Naltrexone
Clozapine	Buprenorphine
First Generation Antipsychotics	Methadone
SSRIs	Antihistamines
SNRIs	Anticholinergics
Bupropion	Atypical Dopaminergic medications
Mirtazapine	
**RECENT MENTAL HEALTH MEDICATIONS** *(24 variables)*<br>*(active prescription within the last 180 days but no prescribed supply extending to Li / VAL start date)*	
Same medications/medication classes as Current Mental Health Medications	

SSRIs = serotonin-specific reuptake inhibitor antidepressants.

SNRIs = serotonin-norepinephrine reuptake inhibitors antidepressants.

TCAs = tricyclic antidepressants.

MAOIs = monoamine oxidase antidepressants.

Appendix 4. Inferences about Residual Confounding Suggested by the Initial Imbalance in the Propensity Score Covariates

The small number of covariates with substantial imbalances prior to matching does not appear to provide, on their own, a clear sense of the likely direction of any residual confounding in our analyses. A quick inspection of Appendix 2, Supplementary Table 1 helps make this point. From this Table, it can be seen that some likely risk factors for suicide death appear more initially more prevalent among patients initiating valproate, while others appear more prevalent among patients initiating lithium. For instance, female patients, who would in general be expected to be associated with lower risk of suicide death (compared to male patients were more prevalent among patients initiating lithium, but black race (which would also be expected to be associated with lower risk of suicide death, compared to white race) was more prevalent among patients initiating valproate. Similarly, expanding the focus beyond simply those covariates with a substantial imbalance to all the covariates presented in Table 1 still provides a mixed picture. For instance, a greater prevalence of patients discharged in the last 7 days prior to treatment initiation, and with suicide attempts requiring nonmental health hospitalizations in the past 30 days, was observed among those patients initiating valproate. A greater prevalence of patients with Bipolar I disorder, however, and with suicide attempts requiring nonmental health hospitalizations in the past 31-180 days, was observed among patients initiating lithium.

Thus, no pattern is evident of such consistency to clearly suggest that the more mentally ill patients, or the patients judged to be of greater imminent suicide risk, were preferentially given one treatment or the other. However, as highlighted in the main manuscript, one piece of information that is particularly important to these judgments is the direction of change in the treatment effect estimate that occurred once <u>all</u> 934 covariates were controlled through matching on the extensive propensity score. Such matching led to a decrease in the degree to which the association between suicide death and lithium was in the direction of greater risks being associated with lithium treatment initiation (0-365 day Odds Ratio [OR] decreased from 1.45 to 1.22). Thus, the overall, combined effect of the factors contained in the entire propensity score covariate set appears to have been to initially bias the effect estimate in the direction of greater risks being associated with lithium initiation than actually attributable to the medication (i.e., some of the risks were due instead to patient characteristics at baseline). Whether or not the remaining, residual confounding biases in this direction is uncertain, but it is certainly plausible that it might, given the suggestion from the data provided by the measured covariates that patients generally at some degree of higher overall risk of suicide death appeared to be preferentially initiated on lithium. This observation is particularly beneficial when combined with the information provided in Table 5 of the manuscript. Manuscript Table 5 indicates that, after propensity score matching, imbalances in the prevalence of coded suicidal ideation existed between the groups in the direction that continues to suggest that patients at higher risk for suicide were preferentially prescribed lithium. Furthermore, if diagnosed suicidal ideation is imbalanced in this direction, it is not implausible that imbalances exist in other, related suicide risk factors with even stronger associations with the risk of suicide death (such as endorsements suicidal planning, intent, and means [12]).

Thus, integrating this information suggests that it is more likely that any residual confounding biased in the direction of associations of observing greater harms from suicide death associated with patients initiating lithium than initiating valproate. Definitive conclusions, however, are not possible, including a judgment of whether this confounding was negligible, modest, or quite significant (and thus whether the associations reported between lithium and suicide death need to be adjusted by a small, medium, or large amount in the direction of protective associations between lithium and suicide death). Clearly additional research is warranted. Nevertheless, we hope that our manuscript both provides a helpful sense of the potential impact of residual confounding and helps spur additional research into the relationship between lithium, suicidal behavior, and suicide death.

Appendix Table 1. Rates of Continuation or Discontinuation of Initial Treatment and Other Censoring Events, by Treatment[a]

Treatment Status	90-day Follow-up				180-day Follow-up				365-day Follow-up			
	Lithium		Valproate		Lithium		Valproate		Lithium		Valproate	
	N	(%)	N	(%)	N	(%)	N	(%)	N	(%)	N	(%)
Still Receiving Initial Treatment	9920	(46.8)	9950	(47.0)	4987	(23.5)	5145	(24.3)	1612	(7.6)	1712	(8.1)
Discontinued Initial Treatment	10455	(49.3)	10858	(51.2)	15146	(71.4)	15532	(73.3)	18344	(86.6)	18873	(89.1)
Initiated opposite mood stabilizer[b]	772	(3.6)	327	(1.5)	992	(4.7)	426	(2.0)	1150	(5.4)	489	(2.3)
Died from Other causes	32	(0.15)	42	(0.20)	52	(0.25)	74	(0.35)	70	(0.33)	99	(0.47)
Died from Suicide	15	(0.07)	17	(0.08)	17	(0.08)	17	(0.08)	18	(0.08)	21	(0.1)

[a] n = 21194 propensity-score matched pairs.

[b] Patients may have reinitiated treatment subsequently with the same or different mood stabilizer, but this occurred after being censored from the "Still Receiving Initial Treatment" subsample of our Intent-to-Treat cohort due to a gap (of ≥15 days) in treatment.

[c] This count provides the number censored due to an immediate switch to the other mood stabilizer.

Appendix 5. Survival Analysis of Suicide Risk by Treatment over 0-90 days, 91-180 days, and 181-365 days

Survival analysis was performed on the intent-to-treat sample using standard Cox regression techniques, but its interpretation was complicated by the fact that "nonproportional hazards" over the 0-365 day time period were observed. The observation of "nonproportional hazards," as evidenced by the crossing of the survival curves at approximately 90 days (Manuscript Figure 2) and a statistically significant time*treatment interaction term (p = 0.03) means that an important assumption of the Cox model was not met. As a response to the observation of nonproportional hazards, we adopted one of several established approaches to addressing nonproportional hazards: segregating follow-up time into periods over which proportional hazards were observed [13].

However, this segregation of time has an important ramification. Some of the follow-up periods have initiation dates after the actual treatment initiation date, which means the close balancing of the treatment groups in the propensity score covariates at day 0 can no longer be presumed to necessarily hold for later time periods. This is a substantial limitation. However, for those readers interested in examining the differences in suicide risk associated with lithium and valproate treatment that accounts for differences in amounts of follow-up time (usually a key function of survival analysis), such results are, for practical purposes, already provided in Tables 3 and 4 of the manuscript. This is because the extensively propensity-score matched treatment groups exhibited highly similar rates of treatment discontinuation for the 0-365 day and briefer time period analyses. For instance, for the primary (intent-to-treat analysis) over 0-365 follow-up time was 7,699, 086 person-days for the lithium treatment group and 7,690,014 person-days for the valproate treatment group (a difference of 0.1%). Thus, analyses that focus on events and patients (logistic regression) provide very similar results to analyses which formally incorporate person-days of exposure, as the very close agreement between the logistic regression and rate ratio results provided in Manuscript Tables 3 and 4 demonstrates.

Given these considerations, we present the logistic regression in the manuscript and the survival analysis results here (Appendix 5 Supplementary Table 1). The survival analysis segregated follow-up time into periods 0-90 days, 91-180 days, and 181-365 days after medication initiation to account for nonproportional hazards. Significantly increased risks of suicide death were observed among all patients initiating lithium, but only for the 91-180 day time period (Hazard Ratio (HR) 3.50, 95% CI 1.41, 8.66; Appendix 5 Supplementary Table 1). Similar to the 0-180 day logistic regression results reported in the manuscript, virtually all elevated suicide risk among patients initiated on lithium during this time period occurred among patients who had stopped or modified lithium treatment (19 out of 21 suicides, HR 3.14, 95% CI 1.25, 7.85). Risks among patients stopping/modifying lithium treatment were less pronounced over other time periods, especially after completion of the first 180 days of follow-up (181-365 HR [after stopping/modifying treatment] 0.93, 95% CI 0.47, 1.80). This lack of increased risks among patients stopping/ modifying treatment over 181-365 days occurred in conjunction with distinctly, although nonsignificantly, reduced risks among patients still receiving initial lithium treatment (HR 0.26, 95% CI 0.03, 2.34, p = 0.23). Although based on extremely few suicides (1 versus 4), these results for active treatment with lithium after 180 days may be consistent with the suggestion that lithium will reduce suicide over longer durations. Such an association could be becoming apparent in our analyses over this period either due to an increasing protective effect of lithium over time or due to diminishment of time-varying confounding in our analyses biasing against lithium.

Given the unusual size of our cohort, to benefit future research we explored whether the nonsignificant association of active lithium treatment after 180 days strengthened if we lengthened follow-up further. Interestingly, the association of lithium with reduced suicide risk among patients still receiving initial treatment strengthened and reached borderline statistical significance from 181–730 days (1 suicide among lithium-treated and 6 suicides among valproate-treated patients, HR 0.18, 95% CI 0.02, 1.45, p = 0.11), but not from 181 days until the end of the study period (up to 10+ years for some patients) (8 suicides among lithium-treated and 8 suicides among valproate-treated patients, HR 1.09, 95% CI 0.41, 2.89). As follow-up time progressively lengthens, the lack of reweighting over time would be expected to be an increasingly strong limitation (that is, the small fraction of patients still receiving lithium after several years of treatment might be considerably different than the fraction of patients continuing to receive valproate). Clearly, still larger cohorts or cohorts with substantially greater rates of treatment persistence will be needed to reliably examine the associations between lithium and valproate treatment and suicide risks over follow-up times longer than 365 days. In addition, alternative designs could be considered to facilitate examinations of longer follow-up times, such as examining the more selectively-reported, but more numerous, outcome of nonfatal suicidal behavior, and using marginal structural models to reweight patient samples during follow-up.

Appendix 5 Supplementary Table 1. Cox Regression Survival Analysis by Time Period since Medication Initiation

Intent-to-Treat Cohort	
Time Period	Hazard Ratio (95% CI)
0-90 days	0.95 (0.60-1.50)[a]
91-180 days	3.50 (1.41-8.66)[b]
181-365 days	0.81 (0.43-1.53)

Stratified by Treatment Status		
Time Period	Hazard Ratio (95% CI)	
	During Exposure to Initial Treatment	After Stopping/Modifying Initial Treatment
0-90 days	0.93 (0.54-1.58)	1.43 (0.24-8.36)
91-180 days	NC[c]	3.14 (1.25 – 7.85)[d]
181-365 days	0.26 (0.03-2.35)[e]	0.93 (0.47-1.84)[f]

[a] Based on Stratified Cox regression (stratified on matched pairs), all other Hazard Ratios non-stratified.

[b] p = 0.007. Other Intent-to-Treat comparisons (0-90 days and 181-365 days were not significant at 0.05 level).

[c] NC = "Not calculable." A hazard ratio cannot be calculated because of 0 suicide deaths in the valproate subcohort still receiving initial treatment over this period. (2 suicide deaths were observed in the lithium subcohort still receiving initial treatment).

[d] p = 0.015. Other comparisons during exposure to initial treatment and after stopping/modifying initial treatment not significant at the 0.05 level.

[e] Based on 5 suicide deaths (1 in lithium and 4 in valproate subcohorts still receiving initial treatment).

[f] Based on 33 suicide deaths (16 in lithium and 17 in valproate subcohorts discontinuing/modifying treatment).

Appendix 6. A Potential Integration of Key Study Findings

Because this study is distinct in its size compared to past studies, it is worthwhile to extract as much information from this study as feasible that may helpfully inform judgments concerning its clinical and research implications. The sections below are intended to provide a succinct summary of the reasons why the study results appear, in a strict sense, to be compatible with four different scenarios: 1) general equivalency between the treatments, 2) increased suicide risks associated with lithium discontinuation, 3) decreased suicide risks associated with active lithium treatment, 4) and, in what we suspect to be the most likely scenario, a combination of some degree of decreased risks during active treatment and some degree of increased risks upon lithium discontinuation. Such a scenario would also appear to be consistent with substantial past literature, given that many nonrandomized studies reporting associations between lithium treatment and reduced risks of suicide were restricted to patients receiving active treatment, and several studies have documented dramatically increased risk of suicide or suicidal behavior shortly after the discontinuation of lithium [14-18]. Nevertheless, it is not clear how much more likely this interpretation is to be true than an interpretation that posits little or no difference between the treatments in their association with suicide risk during active treatment but distinctly differing suicide risks upon discontinuation, or an interpretation that posits substantial decreases in suicide risk associated with active lithium treatment and generally equivalent risks between lithium and valproate upon discontinuation. Given the significant risks observed over the 0-180 and 91-180 days periods (virtually exclusively associated with lithium discontinuation), and the significant differences in diagnostically-coded suicidal ideation (suggestive of bias towards worse outcomes in patients treated with lithium), the final possibility, equivalency between the treatments, appears to be the least likely.

Nevertheless, because this synthesis requires integration of both statistically significant, borderline significant, and (at some points) clearly nonsignificant results, the level of confidence to be placed in this interpretation is highly uncertain. This material should be viewed as a qualitative and nondefinitive synthesis of the overall study findings. It is intended to inform interpretations about the most likely clinical and research implications of this study, without attempting to quantitatively estimate the degree to which these interpretations are more likely than the specific alternatives that are discussed.

Appendix 6A. Implications of Observed High Treatment Discontinuation Rates

The rates of treatment discontinuation observed in the study cohort are quite substantial. In general, the rates of treatment impersistence in this study appear to equal or exceed those reported previously. However, many of these reports do not investigate comprehensive incident cohort samples [19-22]. Three exceptions are the study of Johnson and McFarland [23] examining all patients initiating lithium in an HMO, Kessing et al. [24] examining all patients initiating lithium in Denmark, and the study of Licht et al. [25]. Johnson & McFarland [23] found discontinuation rates slightly greater than our study (median time to discontinuation 72 days, rather than approximately 90 days in our cohort), whereas Kessing et al. found rates slightly lower rates (82% discontinuation in one year rather than the approximately 92% discontinuation in a year observed in our study). Both these studies used estimates of prescription length based on number of pills, rather than using the actual prescription directions to calculate a days' supply as done in this study. In addition, Kessing et al. [24] mentioned their sample might have had lower than average illness severity given that a majority of prescriptions were provided by general practitioners, not psychiatrists. Also, treatment discontinuation rates may be higher in our Veteran sample than other samples, given the high rates of comorbidities, substance use disorders [26], and homelessness. Licht et al. [25] reported only a 19% discontinuation rate for lithium treatment over 2 years; however, their sample was from a specialized lithium clinic and their definition of discontinuation was not provided.

Despite the fact that quite substantial rates of treatment discontinuation are observed, the observed rates are highly similar between the two treatments. For example, at 90 days 46.8% of patients initiating lithium and 47.0% of patients initiating valproate remained on initial treatment, at 180 days 23.5% versus 24.3% patients respectively, and at 365 days 7.6% versus 8.1% of patients, respectively. The fact that rates of discontinuation were highly similar between the two treatment arms is reassuring in one important sense. Given the initial close balance in measured factors, if substantially different treatment discontinuation rates between the treatment groups had been observed instead, this would immediately suggest that the patients remaining on initial treatment in each treatment group differed more substantially on measured suicide risk factors than at initiation. However, while the similarity in rates is reassuring, confirmation that the *reasons* for discontinuation are similar is necessary to firmly conclude that treatment discontinuation occurring during follow-up did not substantially affect the covariate balance between the treatment groups [27]. Such information is often not available [27], especially if the decision to discontinue

treatment (either by the patient or provider) largely depends on factors that are poorly measured or unmeasured (e.g., information about suicidal planning, symptoms such as hopelessness, etc.).

Regardless of whether or not they are factors in decisions to discontinue treatment, concomitant psychiatric medications are one category of measured factors which could potentially exert some degree of influence on this study's findings. This is especially true if concomitant psychiatric medication treatment changes during follow-up. Given that this study's objective was to characterize whether a comparative difference existed in the suicide risk associated with lithium and valproate in their associations with suicide risk among Veterans receiving usual VHA treatment, the study cohort was not restricted to individuals receiving strict monotherapy. Differences between the treatment groups in concomitant psychiatric medications could arise after lithium or valproate initiation, for instance, if a tendency existed for patients in one group to be more likely to add medications during treatment or after initial treatment discontinuation. Because other psychiatric medications may have their own relationship to suicide risk, rigorous examination of these possibilities would ultimately be desirable, and we recommend that this be a focus of future research. However, approaches such as marginal structural models, which periodically reweight samples based on measured factors with the objective of preserving comparability between the treatment groups, may potentially be susceptible to the same amplification of the effects of unmeasured factors as baseline confounding. Therefore, explorations of the effects of concomitant psychiatric medications may need to combine or contrast findings from marginal structural models with approaches that restrict samples to individuals not receiving any concomitant psychiatric medications, or certain psychiatric medications, at any point during follow-up. Because such restrictions reduce sample size, such efforts ideally would involve even larger samples than the one we had available.

It should be recognized that close balancing was achieved of the patient groups on an extensive set of medications psychiatric present at the time of lithium or valproate initiation (Appendix 3), and also on wide variety of other factors. Thus, at least for a substantial portion of the follow-up period (the earlier months), concomitant medications between the groups are likely to be highly similar. In addition, the balance achieved between the treatment groups also included a number of fixed factors (e.g. age) and slowly time-varying factors (e.g. additional psychiatric diagnoses) that might influence prescribing of concomitant medications. Furthermore, a distinct aspect of this study was that not only were current concomitant psychiatric medications controlled, but also the use of psychiatric medications within the last 6 months that were no longer being currently prescribed. To the extent that recent receipt of particular classes of medications might plausibly influence choice concerning what subsequent medications should be initiated, these influences were tightly balanced at baseline. As mentioned above, a design that rebalances the sample during follow-up with respect to concomitant medications is the ideal and should certainly be a future research priority, especially if efforts are made to examine longer periods of follow-up (Appendix 5). This study did incorporate design features which likely limited the impact of selection during follow-up compared to some other designs. However, since we did not formally attempt to control the impacts of selection during follow-up, this limitation should be borne in mind when interpreting this study.

While our study did not attempt a formal analysis incorporating changes occurring during follow-up, some aspects of our study serve to diminish concerns that differences between the treatment groups in the selection of patients to continue or discontinue treatment during follow-up explain all or most of our findings. These include the nature of intent-to-treat estimates, the high rate of treatment discontinuation, and the likely direction of initial confounding.

Differences in selection during treatment alone would not be expected to create the (marginally significant) differences in intent-to-treat estimates measured from treatment initiation. Intent-to-treat estimates continue to incorporate outcomes from all treatment initiators, regardless of a patient's status of still receiving or having discontinued initial treatment. In a sense, patients are not selected out of an intent-to-treat cohort during follow-up. This is especially true for a mortality outcome such as suicide, which is comprehensively documented nationwide, regardless of whether patients continue to receive care from the Veterans Health Administration.

In contrast, substantial differences in selection during follow-up would certainly be expected to bias the treatment effect estimates observed for patients during active treatment (the only type of treatment effect estimate reported in virtually all of the earlier nonrandomized studies of lithium), or after treatment discontinuation. However, if genuine treatment effects were completely absent, such differences in selection would not bias the intent-to-treat estimates, since effects occurring during treatment and after discontinuation are summed. Thus, the marginally statistically significant intent-to-treat results observed associated with lithium treatment after discontinuation over 0-180 days, and the significant risks result over 91-180 days (Appendix 5), are very important observations, since they help restrict the potential explanations for the significantly elevated risk observed in patients discontinuing lithium over 0-180 days.

(Differences in selection certainly may affect intent-to-treat estimates if genuine treatment effects exist, even if these genuine treatment effects are equal between the treatments. Since discontinuation rates were highly similar between treatments, however, differential selection in the context of an equivalent treatment effect generally would be expected to produce a substantial intent-to-treat difference between the treatments only if substantial effect modification was present, even if the reasons for selection in the two groups were different).

The high rates of discontinuation also suggest the possibility that discontinuation decisions may have been made largely by patients rather than providers, and patient-based decisions possibly may have been related much more to concerns such as stigma, side effects, and a lack of a perceived need for treatment than to suicide risk directly. Thus, it is possible that the high rates of discontinuation, while quite sizable, may have had relatively little impact on the treatment effect estimates. In addition, any early provider-based selection that occurred should probably most likely be suspected of occurring in the direction of initial confounding, rather than against it (although this cannot always be assumed to be the case). Initial confounding appears to bias to some degree towards higher risks being associated with lithium, based on the risks observed in the unmatched cohort and the greater prevalence of diagnostically-coded suicidal ideation among patients initiating lithium in the matched cohort. If providers on average selected higher-risk patients to initiate lithium at Day 0, it seems somewhat unlikely that they would reverse this tendency in treatment very shortly after treatment started. Selection in the direction of preferentially retaining higher-risk individuals on lithium than valproate would be in the opposite direction of what would be needed to explain the significantly elevated risks in patients discontinuing lithium over 0-180 days.

As a side note, if selection during follow-up did occur in the direction of retaining higher-risk individuals on lithium, this would represent a likely *third* process that would be expected to lead to an underestimate of any benefit of lithium during active treatment. The other processes likely contributing to this underestimate are, as discussed in the manuscript, residual baseline confounding and the likelihood that some suicides attributed as occurring "during initial treatment" actually occurred after treatment discontinuation.

Appendix 6B. Likelihood of Substantial Residual Confounding Amplification

Propensity score designs, especially when studying rare or infrequent outcomes, permit inclusion of far more covariates than some alternative approaches. We sought to take advantage of this capability to thoroughly control for numerous suicide risk factors and potential suicide risk factors in this study's design by including a large variety of covariates and flexibly modeling their distribution, frequency and timing. We recognized that several potential suicide risk factors (suicidal ideation, planning, etc.) would remain unmeasured. If unmeasured confounding remains uncontrolled in a propensity score analysis, it has recently become appreciated that the effect estimates produced may include an additional source of bias: amplification of whatever confounding remains uncontrolled after application of the propensity score methods [28-31]. This problematic effect would be expected to be increased to the degree that the propensity score includes covariates with a substantial or strong association with treatment exposure in the absence of an association with outcome. We took steps that limit the amount of potential amplification of residual confounding that application of our propensity score approach might produce. All variables were evaluated to determine their relationship with both treatment and outcome. Covariates with particularly substantial relationships with exposure to one or the other treatment were then evaluated individually to assess their plausibility as confounders. Of note, none of the many covariates in the model had what might be considered a particularly "strong" association with treatment exposure by some definitions. That is, no covariates had an odds ratio for treatment exposure to lithium, rather than valproate, of even 3.0 (or 0.33). Nevertheless, given the large number of covariates included in the model, it is possible that some degree of amplification of residual confounding was produced by our design.

However, even if the design created the potential for some amplification of residual confounding, the actual quantitative bias that would result would depend heavily on how much residual confounding was present after application of the propensity score. If little or no residual confounding exists, amplification of this confounding would have to exist on a very pronounced scale (e.g. 2-fold, 3-fold, etc.) to substantially bias the overall findings (assuming a reasonably-sized treatment effect estimate exists). Importantly, with a c statistic of just 0.69, our propensity score is in the lower portion of the range of exposure prediction. A recent simulation, although using R^2 rather than the c statistics, found that propensity scores in the lower portion of the range of exposure prediction should be expected to amplify confounding somewhat modestly (i.e., < 2-fold) [32]. In addition, in our study it remains possible that even the initial confounding may have been fairly minimal, given the generally close balance (standardized difference < 0.10) observed initially for over 98% of the covariates examined.

Nevertheless, we did observe a significant difference in the non-matched covariate denoting the presence of diagnostically-coded suicidal ideation, although the imbalance between treatment groups was only OR = 1.30. Due

to the low prevalence of diagnostically-coded suicidal ideation, this corresponds to a standardized difference of only 0.04, which is still greater than any standardized difference for any variable included in the propensity score (< 0.018). Very little is known about the degree to which diagnostic codes for suicidal ideation underestimates actual suicidal ideation, but underestimation almost certainly occurs. However, sensitivity analyses proportionally boosting the prevalence of suicidal ideation indicates that only when the rates for diagnosed-coded suicidal ideation are multiplied ≥6-fold to reflect possible overall suicidal ideation rates (i.e., including ideation that is both coded and which was not recorded with the diagnostic code) does a standardized difference of ≥ 0.10 occur. This would correspond with suicidal ideation rates in the past 30 days of 15-19%, which may be plausible. This sensitivity exercise also presumes that the same difference in suicidal ideation occurs between the treatment groups for the non-coded suicidal ideation rates as for the diagnostically-coded rates.

The possibility that standardized differences even in an important covariate not included in the propensity score may remain generally modest (e.g., that the standardized difference would remain < 0.10 if the diagnosed suicidal ideation does not underestimate the actual suicidal ideation by six-fold or more) is important. As pointed out above, the influence of confounding amplification on the results is proportional to the amount of residual confounding that remains. If residual confounding is modest, the added effect of residual confounding amplification is likely to be still more modest, at least in this range of exposure prediction [32]. However, just as clearly, if a substantial degree of residual confounding persists (in this study such confounding might result from the known suicide risk factors not able to be incorporated in the model), then amplification of residual confounding could noticeably increase overall residual confounding. In a sense, amplification of residual confounding would be expected to operate similar to amplification in other systems: if residual confounding is minimal, most levels of amplification will not produce a level of confounding that is much different quantitatively. However, if residual confounding is substantial, then amplification of residual confounding can serve to substantially further increase the degree to which the effect estimate reflects confounding.

Perhaps most important observation allaying concerns about at least the most extreme possibilities for residual confounding amplification is the observation that regardless of possible amplification of residual confounding, the propensity score matching methodology appears to have effectively reduced the *overall confounding* observed between the treatment groups. At each time point studied, the odds ratios obtained prior to matching were further from the null than after matching. For instance, over 0-90 days, when the highest proportion of patients were receiving active treatment, matching on measured factors reduced the central estimate of the intent-to-treat odds ratio from 1.10 to 0.95. Over 0-180 days, movement in the intent-to-treat odds ratio estimate from 1.70 to 1.56 was observed after the propensity score matching. Over 0-365 days, the analysis which was informed by the largest number of outcomes, the intent-to-treat odds ratio prior to matching had a central estimate of 1.45, while after matching a central estimate of 1.22 was obtained. Given the number of past findings suggesting that active lithium treatment is associated with either a reduction or at least a neutral association with suicide risk [33, 34], such movement in the estimate away from more extreme increased risks being associated with lithium treatment suggests that overall confounding has been reduced, not amplified.

The observation that overall confounding appears to have been reduced by the propensity score matching methodology does not mean confounding amplification resulting from our methodology does not exist, nor that no confounding exists. Rather, this data suggests that any confounding amplification introduced by our propensity score matching methodology, when added to the remaining confounding already present, is not sufficient to negate the effectiveness of the methodology in improving our reported results by beneficially reducing overall confounding. Of course, if substantial residual confounding amplification is present, this implies that a more optimal control of overall confounding is possible. However, the path to achieving that more optimal state is not necessarily obvious. Removal of variables from the propensity score would certainly be expected to reduce residual confounding amplification, but ironically may increase the amount of residual confounding (if the removed variables actually had a recognized or unrecognized association with outcome), so that overall confounding might actually increase.

The risks of suicide in the unmatched cohort are important to examine for a second reason. They indicate that the general pattern of intent-to-treat risk observed in the matched analysis closely parallels the pattern observed prior to the propensity score matching. That is, the pattern of generally similar intent-to-treat risks over 0-90 days, changing to substantially increased risks with lithium treatment at 0-180 days due to a prominence of risks among patients discontinuing lithium, followed by a lessening of this increased risk at 0-365 days, is not a product of some artefact produced by the propensity score matching. It is a pattern observed even prior to any matching, and thus does not appear to result from the actions of any residual confounding amplification.

Another important observation relevant to judgments about confounding amplification results from the sensitivity analysis in which approximately half of the propensity score variables were removed (Appendix 7). This modification resulted in only a modest change to the effect estimate. Given that removal of these variables were

associated with only a modest change in the treatment effect estimate, the corollary is inclusion of these covariates, despite the fact they exhibited only minimal univariate association with outcome, likely produced only modest confounding amplification. Stated another way, the observation that large risks continue to be associated with lithium treatment discontinuation despite removal of all covariates lacking a substantial association with outcome (+/- 20%) suggests that either risks associated with lithium discontinuation, nonamplified residual confounding, or possibly selection during treatment is largely responsible for those significantly increased risks, rather than amplification of residual confounding. As we discuss in Appendix 6C, this finding does not necessarily mean that overall residual confounding was modest, since we were unable to control for some important risk factors, only that amplification of any residual confounding amplification appears to be modest in effect.

In the future, there are certainly alternative approaches which can be considered when employing an extensive propensity score in a study of suicide risk to potentially optimize confounding control while further limiting confounding amplification. One approach would be to apply an outcome-based selection criteria from the beginning of the study (e.g., such as requiring included covariates have at least a +/-20% association with suicide). In some cases, the lack of a univariate association with outcome does not necessarily indicate that variable is not a genuine confounder. Associations with correlated variables with differing associations with suicide risk could conceal the actual relationship between the variable and the outcome. Alternative approaches might be to adopt the 20% restriction for variables judged particularly unlikely to be associated with suicide risk (e.g., the nonmental health covariates with the least established association with suicide risk), or apply the 20% restriction just to those covariates with the strongest association with treatment, or select the variables on the basis of highly multivariate regression associations. However, the approach which thus far has been demonstrated to apparently minimize confounding thus far in two patient cohorts is a blanket requirement that all covariates have at least a +/- 20% association with outcome [35], although the generalizability of this observation is uncertain. Another decision point to be explored is how to handle multilevel variables. We retained multilevel covariates in which any strata had at least a 20% association with suicide, but alternatives can be readily envisioned of requiring that a majority of strata have at least a 20% association, or all strata have such an association.

Clearly, further research in this area is of particular importance. In sum, however, it does not appear that amplification of residual confounding was likely a major influence upon our findings. This tentative conclusion is suggested by the observations that our overall propensity score approach appeared to result in a substantial reduction in confounding, did not alter the basic pattern of risks over time and by treatment status observed between patients initiating lithium and valproate, and the observation that the removal of almost half of our propensity score covariates had only a modest effect on the treatment effect estimates.

Appendix 6C. Likelihood of Some Residual Confounding Persisting in the Analysis

Although any *amplification* of residual confounding may be modest, at least three lines of evidence that suggests that some degree of residual confounding may persist in the analysis. The first and simplest line of evidence is that rates of suicidal ideation (as reflected by diagnostic codes received by member of the cohort from 2005-2008) were statistically different between the treatment groups. The difference in prevalence in diagnostically-coded suicidal ideation is modest (OR = 1.30, 95% CI 1.09-1.54), and patients who express suicidal ideation are not necessarily those at the highest risk of suicide [36]. However, such patients are almost certainly at higher risk for suicide than many other patients in the cohort, thus this data strongly suggests the presence of at least some degree of baseline confounding biasing against lithium. While it is easy to appreciate how the imbalance between the treatment groups in diagnostically-coded suicidal ideation could potentially reflect residual confounding biasing against lithium, estimating the potential quantitative size of this effect is much more difficult. Nevertheless, such estimates, even if somewhat qualitative, are of considerable importance, given that any degree of residual confounding biasing against lithium suggests a more protective association exists between lithium treatment and suicide risks than estimated from logistic regression.

For instance, Kim et al. found that, from VHA charts of patients receiving treatment for depression, suicidal ideation in the past year in the absence of an attempt was associated with suicide with an odds ratio of approximately 3.0 [12]. If the assumption is made for sensitivity purposes that diagnostically-coded suicidal ideation underestimates actual suicidal ideation by up to a factor of 6, then using the Kim et al. findings would imply that the overall imbalance in suicidal ideation might account for a bias of up to approximately 0.3 on the observed odds ratio. The impact of this imbalance would be less than this amount if it is assumed that diagnostically-coded suicidal ideation rates underestimate genuine suicidal ideation by a factor less extreme than 6-fold; however, the impact of this imbalance could be greater than approximately 0.3 if it is assumed that some of this suicidal ideation was also associated with suicidal planning or preparatory actions acquiring access to means, both of which are more

strongly associated with suicide risk [12]. Thus, it is plausible that the imbalance in suicidal ideation could account for approximately 60% of the increased risk observed among patients stopping/modifying treatment over 365 days (central estimate OR = 1.51), although certainly the impact of suicidal ideation, depending on its prevalence and severity, on observed risk could also be less or more than this amount. Of particular relevance, an impact of this magnitude upon residual confounding resulting from the imbalance of suicidal ideation would imply a central estimate odds ratio during active treatment over 0-365 days of approximately cOR = 0.68, rather than the cOR = 0.86 that was observed.

The second line of evidence is the fact that central effect estimates did change, albeit modestly, during the modified propensity score sensitivity analysis in which almost half the covariates were removed (Appendix 7). This suggests that some degree of confounding amplification may exist, which by extension then implies the presence of some degree of residual confounding still persisting after the propensity score matching. (Some residual confounding must exist for residual confounding amplification to have any noticeable quantitative effect).

The third line of evidence is the least definitive and straightforward, but relates to the observation of increased risks among patients stopping or modifying initial treatment over 0-180 days. Of note, these observed risks both strengthened and remained significant when only patients stopping (rather than stopping, modifying, or resuming treatment) were considered (Manuscript Table 4, Footnote i), when risks were examined among patients stopping or modifying treatment over 91-180 days (Appendix 5). Residual confounding is one of several possible explanations for the observation of increased risk in patients discontinuing one treatment compared to discontinuing another treatment [27, 37]. However, this conclusion is far from definitive because several other processes can influence risk among patients who have stopped initial treatment. In the strictest sense, in order for risks in "former users" to most directly reflect baseline confounding, such confounding must not vary substantially over time, substantial differences must not exist in the rates or reasons for discontinuing treatment between treatment groups [27], any effects from active treatment must not persist into the period after discontinuation,, and/or discontinuation of one medication cannot generate different risks (e.g., "rebound" effects) than discontinuing the comparison medication.

In Appendix 6D, we discuss the evidence from the time course of risk in patients discontinuing treatment that suggests to us that at least some of the risk observed in patients who have discontinued treatment is attributable to risks resulting from discontinuation (or selection), not from confounding. However, in Appendix 6E we will discuss an integrative synthesis that includes a consideration of the associations observed in the intent-to-treat sample and among patients still receiving initial treatment from 181-365 days. These associations suggest not only that an association between active lithium treatment and reduced suicide risk is possible, but also that it may be sizable.

Appendix 6D. Likelihood of Differential Suicide Risk Associated with Lithium versus Valproate Discontinuation

Several lines of evidence appear to support the possibility that differential risk of suicide may be associated the discontinuation of lithium compared to valproate. If so, such differences could explain (along with residual confounding and possibly some contribution from differences in selection during follow-up) part or all of the statistically significant increased risks associated with patients discontinuing lithium compared to valproate over 0-180 days and, in the survival analysis, over 91-180 days.

The most important line of evidence is that the time course of risk appears to more straightforwardly support the possibility of differential risks upon lithium versus valproate discontinuation than residual confounding. If residual confounding was primarily responsible, the general expectation would be that the time-varying pattern of suicide risk over time among patients discontinuing lithium treatment would decrease progressively from a peak in the first 90 days. A pattern of risk consistent with this possibility has been observed in relation to antidepressant initiation [38, 39]. Thus, if the increase in risk observed in those studies over the first 90 days of antidepressant treatment reflects confounding, rather than an iatrogenic effect of antidepressants, then this confounding is most prominent in the first 90 days. However, the possibility cannot be excluded that antidepressants may increase suicidal behavior risk early in treatment in some sensitive individuals, especially of younger age [40].

If initiation of a medication is viewed as a clinical event that likely serves as a marker of a patient sufficiently symptomatic to be at higher than usual risk [39], then it may be relevant to consider the time course of risk concerning other, even more dramatic clinical events which may serve to identify patients as being at particularly high risk (such as suicide attempts and hospital discharges). In these instances, highly time-limited periods (7-30 days) of extreme risk have been observed (i.e., risks of suicide 10-20X greater than what is observed

much later (e.g., 6 months – 1 year subsequently) ([8, 41, 42]. Thus, the general expectation would be that residual confounding, if present, would be the greatest over 0-90 days and decrease in subsequent periods.

Instead, the available data from this study indicates that the difference in risk observed among patients discontinuing lithium compared to valproate is less evident in the first time period (0-90 days) and then becomes much more evident in the subsequent 90 days. (This difference, however, is not statistically significant). This pattern appears more compatible with a developing risk, i.e., a risk that is not initially present but then becomes increasingly present over the first 180 days of treatment. Such an emerging risk fits closely what would be expected from risks among patients discontinuing treatment, in that patients do not start treatment in the status of no longer receiving treatment. Rather, this status must develop over time. Furthermore, the period of highest risk (0-180 days, or more precisely, as the survival analysis suggests, 91-180 days) would incorporate the period of time in which the majority of patients in the cohort would have been discontinued from their treatment for 1-5 months. Interestingly, this corresponds closely to the period previously observed to be of highest risk for mood episode relapse in patients rapidly discontinuing lithium treatment (median time 4.0 +/-0.7 months), although this information was gathered from patients who had generally been receiving long-standing lithium maintenance treatment [43]. Although the time course of the development of risk in the patients discontinuing initial treatment appears very compatible with an emerging risk such as risk associated with discontinuation itself, some caution in interpretation is warranted. The more modest risks observed from 0-90 days in patients discontinuing initial treatment are based only on 5 total suicides, meaning this estimate (which suggests a lower difference in risk among patients discontinuing treatment over the first 90 days than over days 91-180 days) is particularly uncertain.

A second, related line of evidence supports the presence of some degree of differential risk being associated with lithium, compared to valproate, discontinuation. It is important to note that between 0-90 days and 0-180 days the movement in the estimate of risk among patients still receiving initial treatment is modest. A central estimate cOR of 0.88 exists over 0-90 days, compared to a cOR of 1.0 over 0-180 days. (Since there were no suicides in the valproate group on current treatment from 91-180 days, a hazard ratio for this period among patients receiving initial treatment unfortunately is not available. Therefore, comparisons must be made between the 0-90 day and 0-180 day periods, even though one of these periods is inherently nested within the other). If an increase in confounding was the primary or exclusive explanation for why risks in patients discontinuing treatment increased from 1.49 (central estimate) over 0-90 days to 2.72 over 0-180 days (quite a sizeable central estimate increase), then such confounding would be expected to also increase the apparent risks associated with active treatment considerably (unless the treatment effect strengthened quickly). Some minor increase in risk associated with active treatment does occur, but nothing similar in size to the increase occurring among patients discontinuing treatment. This suggests that the increase in patients discontinuing lithium treatment most likely not due primarily to increases in time-varying confounding. This pattern also suggests the elevated risks are not the product of selection during follow-up favoring the highest risk individuals being discontinued from lithium. While such selection would produce increased risk of suicide being observed in conjunction lithium, rather than valproate, discontinuation, it would also be expected to result in a compensatory *decrease* in risk in patients still receiving active treatment. Instead, the risk increases slightly (from central estimate cOR = 0.88 to central estimate cOR = 1.0). Thus, the relative stability observed in the estimate of suicide risk associated with the two treatments among patients still receiving initial treatment between 0-90 and 0-180 days suggests that the differing risks of suicide observed upon lithium, compared to valproate, is most easily explained by a process that would be restricted just to the patients discontinuing initial treatment. The two other candidates to influence this effect estimate, confounding and selection during treatment, both would be expected to substantially affect the treatment effect estimate for patients still receiving active treatment as well. Thus, by process of elimination, this data most easily supports the existence of risks being associated directly with the discontinuation of lithium, compared to valproate. However, how much more likely this possibility is than the alternatives of confounding and selection during treatment cannot be determined, and complex combinations of two or three of these processes occurring simultaneously cannot be excluded.

The third line of evidence is that the risk appears to completely resolve by 181-365 days (although random variation could contribute to this finding) (Appendix 5). If confounding increased over 91-180 days compared to 0-90 days, it seems appear less plausible this confounding would virtually completely resolve by 181-365 days. However, a rapid resolution of risk is more plausible if the peak risks directly associated with lithium discontinuation were highly time-limited (as has been somewhat observed for the risk of mood episode relapse) [43]. As discussed in Appendix 5, very few suicides occurred in patients "newly discontinuing" over 181-365 days, thus the risk estimate for patients stopping/modifying initial treatment are particularly influenced by the risk observed among the largest segment, by far, of patients are counted as having stopped/modified treatment: those who initially discontinued months ago (i.e., over 0-180 days). Previous studies of suicide risks after discontinuation of lithium maintenance treatment have found the increased risk to be clearly time-limited, although the analyses do not address

whether the time-limited period of risk is confined to any time period shorter than the first year after discontinuation [18]. However, it is especially notable that the Goodwin et al. 2003 cohort study [44], which, like this one examined risks starting at the point of treatment initiation, noted that 32% of all the suicides occurring after treatment discontinuation occurred within the first month after discontinuation. Unfortunately, the Goodwin et al. study [44] did not report whether this suicide risk differed between lithium, valproate, and/or carbamazepine (perhaps because the low numbers likely would have prevented any statistical significance findings). Nor did their report describe whether these risks occurred in conjunction with discontinuation occurring early or late in treatment.

One caution for this interpretation is that the two previous studies by Yerevanian and colleagues which compared risk of discontinuing lithium and valproate found discontinuation of both medications to be associated with similar and substantial increased risks of suicidal events (suicidal behavior or hospitalization for suicidal ideation) [17, 45]. One major potential difference between the studies, however, is that our study was focused exclusively on risks observed within one year of initiation, while the Yerevanian studies typically examined lengthy courses of treatment, on average. For example, average follow-up in the 2007 study was approximately 38 months per patient, of which approximately > 90% of this time was accounted for by time receiving medication. This longer follow-up time, including more time on medication further from medication initiation, might have served to strengthen the association of both medications with relatively low rates of suicide during treatment. This in turn may have produced a larger contrast in suicide risks upon discontinuation for both medications. This would be especially true if the discontinuation is preceded by psychiatric decompensation that prompted either the patient or provider to discontinue treatment.

Appendix 6E. Summary and Integration of Key Findings

Although initially our data may appear most straightforwardly consistent with an interpretation that lithium treatment is associated with either similar suicide risks or increased suicide risks compared to valproate in this Veterans cohort over the first 365 days of treatment, several key complexities present themselves. The first complexity is that any increased risk associated with lithium treatment appears to be entirely or almost entirely associated with risks observed after treatment discontinuation, not during active treatment. This suggests that, in contrast to most comparative effectiveness studies, the degree to which the medications may differ in effectiveness relates substantially to what is observed after treatment discontinuation, rather than during treatment.

The second major complexity is the highly time-varying pattern of the intent-to-treat risks, going from a central estimate hazard ratio of 0.95 at 0-90 days to 3.50 at 91-180 days to 0.81 at 181-365 days, with the 91-180 day hazard ratio being statistically significant. This pattern, although it could reflect a large contribution from random variation, appears suggestive of a substantial emergent risk developing after 90 days of treatment in those discontinuing treatment. In general, baseline confounding occurring after a marker of high risk such as treatment initiation [39] would be expected to diminish steadily, with the highest risk being observed shortly after treatment initiation (Appendix 6D). Therefore, residual confounding does not appear to be a good candidate to explain this emergent risk, nor does another possibility, selection during follow-up. In theory, selection of patients during follow-up could certainly produce such an emergent risk in patients discontinuing lithium, if the patients being discontinued from lithium were those at particularly high risk for suicide. However, in the absence of a genuine medication effect, selection during follow-up alone, in two treatment groups with similar discontinuation rates, would not be expected to likely alter intent-to-treat risks measured from treatment initiation (Appendix 6A). Although we observe only marginally significant intent-to-treat risks from initiation over 180 days, it also should be noted that if selection was occurring in the direction to explain the elevated risks among patients discontinuing lithium at 180 days, this selection should also engender a reduction in risk among patients remaining on initial treatment (since the highest risk individuals are being removed from this patient group). This is not what is observed, instead, the central estimate of the risk among patients still receiving initial treatment stays essentially the same (to be precise, increases slightly, rather than decreases). If confounding or selection during follow-up is not accounting for this sharply increased risk at 91-180 days, then the emergent risk that is suggested is suicide risk that is associated with the discontinuation of lithium early during lithium treatment. Furthermore, it would appear that such risk is somewhat limited to a relatively brief period after lithium discontinuation, seemingly similar to the timing of risks for mood episode recurrences previously noted by others after rapid discontinuation of lithium treatment [43].

The third major complexity is that intent-to-treat risks do not remain substantially elevated in the final time period, but rather decrease to such an extent that an intent-to-treat estimate is in the direction of lower suicide risk associated with lithium for 181-365 days (central estimate hazard ratio [HR] = 0.81). This finding is clearly nonsignificant, and thus potentially being the result of chance. Nevertheless, although the role of chance limits the

weight that can be placed on this finding, this is potentially a very important observation, given that both any residual confounding and risks associated with discontinuation appear clearly to be most likely associated in the direction of greater suicide risk observed with lithium. If both these important components of an intent-to-treat estimate would be expected to be in the direction of increased risk being associated with lithium treatment, then an obvious candidate that remains to account for a reduction in intent-to-treat suicide risks is an active medication effect among the patients still receiving initial treatment. The observed risks over 181-365 days also suggest that both any confounding and the effects of risk from discontinuation for the bulk of the cohort have resolved, a conclusion consistent with the HR = 0.93 observed among patients who have discontinued treatment.

The opportunity to examine risks from this period (181-365 days after initiation) are of particular interest, not only because of the possibility for observing relatively unconfounded estimates of lithium's treatment effect that the data somewhat suggests, but also because of the potential size of that possible effect. The reduced risk estimated by the central estimate of the intent-to-treat hazard ratio for this time period (HR = 0.81) is not clinically insubstantial, although it must be kept in mind that this association does not achieve statistical significant and part of this reduction appears accounted for by the slightly reduced risk of suicide (for this particular period) in patients who have discontinued lithium, compared to valproate, treatment (the large majority of which would now would be separated by months from their discontinuation event). Nevertheless, this observation suggests there is at least a reasonable possibility that active lithium treatment is serving to reduce suicide risks of the fraction of patients still receiving lithium within this time period. Given that active treatment now only represents about 16% of the total follow-up time contributing to the intent-to-treat estimate within this time period, to the extent that the effects of active treatment are contributing to this intent-to-treat effect estimate, the association between active lithium treatment and reduced suicide risk could be rather sizeable. Consistent with this inference, a sizable association (HR = 0.26, 95% CI 0.03, 2.35) is what is observed among patients still receiving their initial treatment, although it does not reach statistical significance (p = 0.23), is informed by just 5 suicides total (1 in lithium recipients and 4 in valproate recipients), and may also reflect contributions from any effects of differential selection during follow-up. Thus, despite the overall nonsignificance of our primary analysis over 0-365 days, and the significant associations observed between lithium discontinuation and increased suicide risk over the first 180 days, our data also suggests, although with much less confidence, that a clinically meaningful reduction in suicide risk may be associated with active lithium treatment after just 181 days of treatment. In this context, it is noteworthy that when the analysis of individuals still receiving initial treatment is continued to 730 days, this association strengthens (HR = 0.18) and almost achieves marginal statistical significance (p = 0.11) (Appendix 5), although this relationship does not persist until the end of follow-up.

It should also be noted that, because of the direction of any residual confounding apparently biases against lithium, it is plausible that similar reductions in suicide risk exist over 0-180 days, but this reduction is simply concealed by confounding. In addition, as mentioned in Appendix 6A, it is plausible that if selection early during treatment paralleled initial confounding, then selection during follow-up may have also biased against finding associations between lithium and reduced suicide risk during active treatment. In a sense, this study may provide a useful contrast to earlier literature in which baseline confounding was expected to be in the direction of finding an association between lithium and decreased suicide risk [46]. If selection during follow-up generally paralleled initial confounding in this early literature, associations between lithium and suicide risk would be expected to overstate lithium's benefits. The additional importance of the direction of baseline confounding in predicting both the direction of bias to the effect estimates from confounding, but also potentially from selection at least early during treatment, reinforces the particular value that would arise if cohorts with apparently minimal confounding could be identified and examined in for at least some of the future nonrandomized studies of lithium and suicide risk.

Given that the likely direction of any residual confounding, and potentially selection during follow-up, biases against observing any association between lithium and reduced suicide risk, this study is more likely to underestimate than overestimate the benefits of active lithium treatment. This aspect of this study should be kept in mind when comparing this study to other literature.

Appendix 6F. Recommendations for Clinical Practice Emerging from This Study's Results

This qualitative integration of the evidence from our study supports a number of important clinical and research recommendations. First, the clearest findings from our study related to the statistically significantly increased risk of suicide among patients discontinuing lithium over the first 180 days of the study. Although these risks are in the likely direction of any residual confounding, the distinct time course of their emergence strongly suggest the presence of at least some degree of increased risk being associated with lithium, compared to valproate, discontinuation. These findings indicate that patients should be warned about the possibility of experiencing an

increased risk for suicide should they choose to discontinue their treatment, and that providers should also be educated concerning this possible unintended consequence of lithium treatment. In general, persistence with lithium treatment once initiated should be maximized if possible and clinically appropriate. Maximizing persistence may have the dual benefit of maximizing any beneficial associations of active lithium treatment with reduced suicide risk and minimizing the risks associated with lithium discontinuation. Useful reviews of evidence-based approaches to maximize adherence to mood stabilizers have been published [47]. Providers should also be educated that, should discontinuation prove necessary, gradual, rather than rapid, discontinuation of lithium should be implemented when clinically appropriate. Gradual discontinuation appears to substantially reduces the risk of mood episode relapse [43, 48] and thus plausibly may also decrease any associated suicide risk. (However, the possibilities that this difference in risk may relate all or in part to the characteristics of patients able to discontinue gradually versus those not able to discontinue gradually cannot be currently ruled out). Patients who do discontinue treatment should also be educated to monitor themselves closely, and providers should monitor such patients closely when feasible. Such monitoring is already recommended in general after mood stabilizer discontinuation [49].

In addition, this study provides several important research recommendations, which are discussed further in Appendix 8.

Appendix 7. Modified Propensity Score Analysis

As part of the evolution of propensity score methods, concerns have been raised that inclusion of variables that are not strongly related to outcome may actually increase the impact of confounders not included in the analysis [31, 32]. Procedures for handling this possibility have been debated, but one approach that has been evaluated in the literature has been to restrict the propensity score simply to variables with a strong association with outcome (e.g. +/-20 %) [35]. We applied this approach to our data in an exploratory analysis focused on the time period with the most statistically significant findings (0-180 days). All covariates associated with a univariate OR with suicide of between 0.83-1.19 were removed from the propensity score (approximately 50% of the total number of covariates). Because of the interest in risk in patients stopping treatment entirely, rather than simply modifying treatment, for this exploratory analysis we removed patients who modified or resumed their treatment to obtain a "no longer exposed" sample of follow-up time from patients restricted to those who discontinued treatment. The following results were obtained, compared to the results for this analysis for the full propensity score (given in Table 4, Footnote i, and below).

Full Propensity Score Analysis:

Patients Still Receiving Initial Treatment (0-180 days): Conditional Odds Ratio (cOR) 1.00, 95% CI 0.51-1.96; Rate Ratio: 1.01

Patients No Longer Exposed (i.e., removing patients who modify or discontinue and subsequently resume treatment) (0-180 days): Odds Ratio (OR): 3.61, 95% CI 1.34-9.73, Rate Ratio 3.60

Modified Propensity Score Analysis (removing variables not associated with a +/-20% change in the odds of suicide from the propensity score):

Patients Still Receiving Initial Treatment (0-180 days): cOR 1.00; 95% CI 0.58-1.72; Rate Ratio 1.22

Patients No Longer Exposed (0-180 days): OR 3.00; 95% CI 1.19-7.55; Rate Ratio 2.98

This analysis provides suggestive evidence that the overall contribution of any amplification of confounding to the effect estimates may be relatively modest (given that only a 24% change in risks among patients discontinuing treatment is observed after this substantial change in the propensity score was executed). Such a finding appears consistent with other lines of evidence suggesting that residual confounding amplification does not overly impair this analysis (Appendix 6B). The development of methodology for assessing the possibility of confounding amplification, however, is still embryonic. That consideration, plus the large role that may be played by statistical uncertainty given the wide confidence intervals in this study, means definitive conclusions about the amount of residual confounding amplification cannot be reached. Since propensity score methods are specifically sensitive to this potential effect, the possibility of residual confounding amplification should be kept in mind in the interpretation of this study's results and during comparisons to previous findings.

Appendix 8. Suggestions Concerning Future Research

Despite this study's unprecedented size, one fundamental conclusion of the study is that further research concerning the associations between lithium and suicide risk needs to be vigorously conducted. Even without this study, differences between recent and past randomized and nonrandomized research suggest that questions remain concerning the degree to which lithium treatment may be associated with reductions in suicide and suicidal behavior risk. Furthermore, there is an increasing awareness among healthcare researchers that nonrandomized studies may easily contain confounding bias related to the characteristics of those patients, even within specific diagnostic categories, that are chosen to initiate one medication compared to another, despite efforts to rigorously control for measurable patient differences. Mental health research may be particularly sensitive to this potential confounding. As we indicate, despite the extensiveness of our covariates, like virtually all of its predecessors this study lacks extensive information concerning several potential confounders such as suicidal ideation, planning and means, psychiatric symptoms, and recent stressors.

Nevertheless, our study, through its inclusion of intent-to-treat and post-discontinuation risk estimates, will hopefully serve to help focus future mental health research into the question of lithium and suicide risk. First, this study has indicated that increased investigative focus should be placed on examining the possibility that lithium treatment, in cohorts with very high discontinuation rates, might actually increase overall (intent-to-treat) risks of suicide in the short term (if it is determined that lithium discontinuation does indeed pose greater acute suicide risks than valproate discontinuation). Second, however, our results remain compatible with the possibility that active treatment with lithium may be associated with substantial reductions in suicide risk. This also should be the focus of energetic follow-up research, especially since relatively few effective interventions against suicides are known, including among medications. Consider, for instance, the possibility that sufficient residual confounding and/or selection during follow-up biasing against lithium might persist in our analysis to conceal a protective association between lithium and suicide death similar to that observed for clozapine (HR = 0.76) for suicidal events. In this case, lithium would likely be a much more valuable intervention, given that lithium would be exhibiting such as effect size related to suicide death, not suicide events (a reduced risk of suicide death was not observed for clozapine [50]), lithium's potential use across a broader range of psychiatric diagnoses, and its much less burdensome monitoring requirements.

This study has reemphasized the need to become even more rigorous about attempting to control for confounding at baseline, given that imbalances appear to persist in the factors (e.g., diagnostically-coded suicidal ideation) not able to be included in the propensity score (despite very tight balance being achieved in numerous other covariates). Such additional research could take several forms. Randomized trials, although challenging to execute, would undoubtedly provide the most rigorous, unconfounded answer regarding the effectiveness of lithium and comparison medications against suicidal behavior, if such trials can be conducted practically (numerous participants would be needed), safely (recommendations how to do so have been advanced) [50, 51], and ethically (i.e., through comparisons with genuine equipoise). Instrumental variables such as prescriber preference variables [52] may also be valuable to investigate, given the potential capability of instrumental variables to balance unmeasured factors. Not all the assumptions underlying instrumental variable analysis, however, can be rigorously tested. Nevertheless, in other treatment studies in which unmeasured confounding was suspected, instrumental variables produce effect estimates closer to those obtained by randomized trials than propensity score methods [53]. Chart review study designs [54], potentially combined with marginal structural models to address changes in medication, risk factor, and suicidal ideation changes during follow-up, would likely constitute a useful enhancement in cohorts with substantial discontinuation of treatment.

Addressing factors such as suicidal ideation, planning, and means, recent stressors, and recent or current psychiatric symptoms will not be simple, and likely will entail potentially laborious manual chart reviews unless effective automated methods to identify these factors can be developed. It seems likely that case-control or case-cohort designs may need to be adopted to reduce the total number of charts to be reviewed to a feasible number.

In addition, research is needed into how to optimize selection strategies for variables in propensity scores to maximize their benefits in reducing confounding while minimizing potential confounding amplification. This would be especially valuable for studies of suicide risk, since suicide risk is sufficiently multifactorial that approaches such as propensity scores using extensive covariates will likely continue to be desirable. Finally, our study has illustrated the importance of subsequent research adopting methodology such as marginal structural models that will help facilitate examination of outcomes over longer follow-up periods. Studies over longer follow-up could prove useful for two reasons. First, the associations between lithium or comparison medications and suicide risk may strengthen or weaken over time. Second, if baseline confounding is substantially time-varying, periods later in follow-up may

have less confounding, as some of our findings suggest.*

However, while chart review approaches might provide improved information about suicidal ideation, psychiatric symptoms, and stressors, such information would almost certainly be still incomplete. For this reason, among nonrandomized studies either instrumental variable analysis (as mentioned above) is likely to be particularly valuable, or cohort studies or nested case control studies from cohorts large enough and with sufficient adherence that substantial numbers of patients continue to receive initial treatment for more than just 6 months – 1 year after initiation. It is plausible that if patients were directed to one medication or the other on the basis of suicide risk initially, any time-varying components to this risk may have largely resolved by that point. Rebalancing on measured factors at that point (i.e. 6 months or one year after initiation) and starting follow-up might be one approach to particularly limit confounding in nonrandomized studies of lithium and comparison medications. The advantages of a patient sample that might be largely devoid of confounding might outweigh concerns that the results would be the most strictly generalizable for the rather select population of patients who are adherent to the medications for 6 months or more.

Large cohort studies may also provide other valuable information. Cohorts with greater adherence in general would provide greater power to detect any reductions in suicide risk associated with active lithium treatment (although power to examine risks in patients discontinuing lithium would then be lessened) [27]. Research in some international settings for which lithium treatment remains more routine also may potentially yield lower levels of baseline confounding than studies in the United States, in which only a decided minority of patients receive lithium. As studies become more sophisticated and as sample sizes continue to increase, consideration should be given to incorporating information both about dose and compliance based on additional information besides prescription records (e.g., serum blood levels). Regarding dose, one approach might be to categorize patients into those receiving "high dose" (i.e. equal or above the median dose) or "low dose" treatment. Such strata may become complicated to define as patients shift from one status to another over time, although perhaps this could be reflected in marginal structural models or similar approaches. Ideally, judgments concerning dose should take into account a patients' age and renal function (and possibly weight), since lower doses are routinely and appropriately used in older patients. As an extreme, formulas exist to calculate expected lithium serum levels based on renal function and other factors, but it is uncertain how valuable this level of precision may be, given that patient fidelity with dosing recommendations usually cannot be ascertained. Serum blood levels can reflect adherence at certain points, but it is unclear how often that determination will be made close to a point of clinical interest (i.e., an outcome such as suicide or suicidal behavior). In some unusually large analyses, sufficient numbers of patients may exist to permit an examination of only those patients with serum blood level-documented adherence or nonadherence to treatment. Otherwise, serum blood level information may have to be used more qualitatively to determine whether patients appear to have histories of good or poor treatment persistence in the study.

Given the possibility that lithium may increase suicide risks upon discontinuation for some period of time, nonrandomized research should also strive to incorporate an intent-to-treat perspective that ascertains outcomes for individuals both receiving and no longer receiving their initiated treatment. Such an approach will also help facilitate comparisons to randomized research.

It is hoped that the study reported here will help contribute to continued improvements in the investigation of the associations of lithium, and other psychiatric medications, with suicide death and suicidal behavior. The question of whether psychiatric treatments are associated with altered risks of suicide death or suicidal behavior is clearly of the utmost importance to patients and providers alike.

* Because marginal structural models and other approaches allow a sample to be periodically rebalanced in risk factor composition over time, they may also prove valuable for investigating one further finding from our study. Although not statistically significant and therefore potentially incidental, the finding of numerically lower suicide rates in the valproate cohort after discontinuation of valproate than during valproate treatment (Table 3, and Table 4, 0-180 day results) deserves further investigation given the US Food and Drug Administration labeling warning concerning the possibility of increased suicidal ideation or behavior during anticonvulsant treatment.

References

1. Lauterbach E, Felber W, Muller-Oerlinghausen B, Ahrens B, Bronisch T, Meyer T, Kilb B, Lewitzka U, Hawellek B, Quante A *et al*: **Adjunctive lithium treatment in the prevention of suicidal behaviour in depressive disorders: a randomised, placebo-controlled, 1-year trial**. *Acta Psychiatr Scand* 2008, **118**(6):469-479.
2. Guzzetta F, Tondo L, Centorrino F, Baldessarini RJ: **Lithium treatment reduces suicide risk in recurrent major depressive disorder**. *J Clin Psychiatry* 2007, **68**(3):380-383.
3. Schneeweiss S, Rassen JA, Glynn RJ, Avorn J, Mogun H, Brookhart MA: **High-dimensional propensity score adjustment in studies of treatment effects using health care claims data**. *Epidemiology* 2009, **20**(4):512-522.
4. Toh S, Garcia Rodriguez LA, Hernan MA: **Confounding adjustment via a semi-automated high-dimensional propensity score algorithm: an application to electronic medical records**. *Pharmacoepidemiol Drug Saf* 2011, **20**(8):849-857.
5. Shack LG, Rachet B, Williams EM, Northover JM, Coleman MP: **Does the timing of comorbidity affect colorectal cancer survival? A population based study**. *Postgrad Med J* 2010, **86**(1012):73-78.
6. Harris EC, Barraclough B: **Suicide as an outcome for mental disorders. A meta-analysis**. *The British journal of psychiatry : the journal of mental science* 1997, **170**:205-228.
7. Quan H, Sundararajan V, Halfon P, Fong A, Burnand B, Luthi JC, Saunders LD, Beck CA, Feasby TE, Ghali WA: **Coding algorithms for defining comorbidities in ICD-9-CM and ICD-10 administrative data**. *Med Care* 2005, **43**(11):1130-1139.
8. Haukka J, Suominen K, Partonen T, Lonnqvist J: **Determinants and outcomes of serious attempted suicide: a nationwide study in Finland, 1996-2003**. *Am J Epidemiol* 2008, **167**(10):1155-1163.
9. Kapur N, Cooper J, King-Hele S, Webb R, Lawlor M, Rodway C, Appleby L: **The repetition of suicidal behavior: a multicenter cohort study**. *Journal of Clinical Psychiatry* 2006, **67**(10):1599-1609.
10. Kim HM, Smith EG, Stano CM, Ganoczy D, Zivin K, Walters H, Valenstein M: **Validation of key behaviourally based mental health diagnoses in administrative data: suicide attempt, alcohol abuse, illicit drug abuse and tobacco use**. *BMC Health Serv Res* 2012, **12**:18.
11. Center for Disease Control: National Center for Injury Prevention and Control. **WISQARS Injury Mortality Reports, 1999-2007** [http://webappa.cdc.gov/sasweb/ncipc/mortrate10_sy.html]. Accessed October 29, 2014.
12. Kim HM, Smith EG, Ganoczy D, Walters H, Stano CM, Ilgen MA, Bohnert AS, Valenstein M: **Predictors of suicide in patient charts among patients with depression in the Veterans Health Administration health system: importance of prescription drug and alcohol abuse**. *The Journal of clinical psychiatry* 2012, **73**(10):e1269-1275.
13. Parikh NI, Gona P, Larson MG, Wang TJ, Newton-Cheh C, Levy D, Benjamin EJ, Kannel WB, Vasan RS: **Plasma renin and risk of cardiovascular disease and mortality: the Framingham Heart Study**. *Eur Heart J* 2007, **28**(21):2644-2652.
14. Nilsson A: **Lithium therapy and suicide risk**. *J Clin Psychiatry* 1999, **60 Suppl 2**:85-88; discussion 111-116.
15. Muller-Oerlinghausen B, Muser-Causemann B, Volk J: **Suicides and parasuicides in a high-risk patient group on and off lithium long-term medication**. *J Affect Disord* 1992, **25**(4):261-269.
16. Yerevanian BI, Koek RJ, Feusner JD: **Pharmacotherapy and risk of suicidal behaviors among patients with bipolar disorder**. *JAMA* 2004, **291**(8):939; author reply 940.
17. Yerevanian BI, Koek RJ, Mintz J: **Bipolar pharmacotherapy and suicidal behavior. Part I: Lithium, divalproex and carbamazepine**. *J Affect Disord* 2007, **103**(1-3):5-11.
18. Baldessarini RJ, Tondo L, Hennen J: **Effects of lithium treatment and its discontinuation on suicidal behavior in bipolar manic-depressive disorders**. *J Clin Psychiatry* 1999, **60 Suppl 2**:77-84; discussion 111-116.
19. Schumann C, Lenz G, Berghofer A, Muller-Oerlinghausen B: **Non-adherence with long-term prophylaxis: a 6-year naturalistic follow-up study of affectively ill patients**. *Psychiatry Res* 1999, **89**(3):247-257.
20. Scott J, Pope M: **Self-reported adherence to treatment with mood stabilizers, plasma levels, and psychiatric hospitalization**. *Am J Psychiatry* 2002, **159**(11):1927-1929.
21. Wolfsperger M, Greil W, Rossler W, Grohmann R: **Pharmacological treatment of acute mania in psychiatric in-patients between 1994 and 2004**. *J Affect Disord* 2007, **99**(1-3):9-17.

22. Svarstad BL, Shireman TI, Sweeney JK: **Using drug claims data to assess the relationship of medication adherence with hospitalization and costs**. *Psychiatr Serv* 2001, **52**(6):805-811.
23. Johnson RE, McFarland BH: **Lithium use and discontinuation in a health maintenance organization**. *Am J Psychiatry* 1996, **153**(8):993-1000.
24. Kessing LV, Sondergard L, Kvist K, Andersen PK: **Adherence to lithium in naturalistic settings: results from a nationwide pharmacoepidemiological study**. *Bipolar Disord* 2007, **9**(7):730-736.
25. Licht RW, Vestergaard P, Rasmussen NA, Jepsen K, Brodersen A, Hansen PE: **A lithium clinic for bipolar patients: 2-year outcome of the first 148 patients**. *Acta Psychiatr Scand* 2001, **104**(5):387-390.
26. Manwani SG, Szilagyi KA, Zablotsky B, Hennen J, Griffin ML, Weiss RD: **Adherence to pharmacotherapy in bipolar disorder patients with and without co-occurring substance use disorders**. *J Clin Psychiatry* 2007, **68**(8):1172-1176.
27. Hernan MA, Robins JM: **Authors' response, part I: observational studies analyzed like randomized experiments: best of both worlds**. *Epidemiology* 2008, **19**(6):789-792.
28. Bhattacharya J, Vogt W: **Do instrumental variables belong in propensity scores?** *NBER Technical Working Paper no 343.* Cambridge, MA: National Bureau of Economic Research, 2007. [http://www.nber.org/papers/t0343]. Accessed October 29, 2014.
29. Wooldridge J: **Should instrumental variables be used as matching variables?** East Lansing, MI: Michigan State University; 2009. [http://econ.msu.edu/faculty/wooldridge/docs/treat1r6.pdf]. Accessed October 29, 2014.
30. Pearl J: **On a class of bias-amplifying variables that endanger effect estimates**. In: *Proceedings of the Twenty-Sixth Conference on Uncertainty in Artificial Intelligence (UAI 2010); Corvallis, OR*: Association for Uncertainty in Artificial Intelligence: 2010: 2425-2432.
31. Pearl J: **Invited commentary: understanding bias amplification**. *Am J Epidemiol* 2011, **174**(11):1223-1227; discussion pg 1228-1229.
32. Brooks JM, Ohsfeldt RL: **Squeezing the balloon: propensity scores and unmeasured covariate balance**. *Health Serv Res* 2013, **48**(4):1487-1507.
33. Baldessarini RJ, Tondo L: **Lithium and suicidal risk**. *Bipolar Disord* 2008, **10**(1):114-115.
34. Cipriani A, Pretty H, Hawton K, Geddes JR: **Lithium in the prevention of suicidal behavior and all-cause mortality in patients with mood disorders: a systematic review of randomized trials**. *Am J Psychiatry* 2005, **162**(10):1805-1819.
35. Patrick AR, Schneeweiss S, Brookhart MA, Glynn RJ, Rothman KJ, Avorn J, Sturmer T: **The implications of propensity score variable selection strategies in pharmacoepidemiology: an empirical illustration**. *Pharmacoepidemiology and Drug Safety* 2011, **20**(6):551-559.
36. Smith EG, Kim HM, Ganoczy D, Stano C, Pfeiffer PN, Valenstein M: **Suicide risk assessment received prior to suicide death by Veterans Health Administration patients with a history of depression**. *The Journal of clinical psychiatry* 2013, **74**(3):226-232.
37. Cooper WO, Habel LA, Sox CM, Chan KA, Arbogast PG, Cheetham TC, Murray KT, Quinn VP, Stein CM, Callahan ST *et al*: **ADHD drugs and serious cardiovascular events in children and young adults**. *N Engl J Med* 2011, **365**(20):1896-1904.
38. Jick H, Kaye JA, Jick SS: **Antidepressants and the risk of suicidal behaviors**. *JAMA* 2004, **292**(3):338-343.
39. Valenstein M, Kim HM, Ganoczy D, McCarthy JF, Zivin K, Austin KL, Hoggatt K, Eisenberg D, Piette JD, Blow FC *et al*: **Higher-risk periods for suicide among VA patients receiving depression treatment: prioritizing suicide prevention efforts**. *J Affect Disord* 2009, **112**(1-3):50-58.
40. Stone M, Laughren T, Jones ML, Levenson M, Holland PC, Hughes A, Hammad TA, Temple R, Rochester G: **Risk of suicidality in clinical trials of antidepressants in adults: analysis of proprietary data submitted to US Food and Drug Administration**. *BMJ* 2009, **339**:b2880.
41. Qin P, Agerbo E, Mortensen PB: **Suicide risk in relation to socioeconomic, demographic, psychiatric, and familial factors: a national register-based study of all suicides in Denmark, 1981-1997**. *Am J Psychiatry* 2003, **160**(4):765-772.
42. Goldacre M, Seagroatt V, Hawton K: **Suicide after discharge from psychiatric inpatient care**. *Lancet* 1993, **342**(8866):283-286.
43. Baldessarini RJ, Tondo L, Faedda GL, Suppes TR, Floris G, Rudas N: **Effects of the rate of discontinuing lithium maintenance treatment in bipolar disorders**. *J Clin Psychiatry* 1996, **57**(10):441-448.
44. Goodwin FK, Fireman B, Simon GE, Hunkeler EM, Lee J, Revicki D: **Suicide risk in bipolar disorder during treatment with lithium and divalproex**. *JAMA* 2003, **290**(11):1467-1473.

45. Yerevanian BI, Koek RJ, Mintz J: **Lithium, anticonvulsants and suicidal behavior in bipolar disorder**. *J Affect Disord* 2003, **73**(3):223-228.
46. Ernst CL, Goldberg JF: **Antisuicide properties of psychotropic drugs: a critical review**. *Harv Rev Psychiatry* 2004, **12**(1):14-41.
47. Lolich M, Vazquez GH, Alvarez LM, Tamayo JM: **Psychosocial interventions in bipolar disorder: a review**. *Actas espanolas de psiquiatria* 2012, **40**(2):84-92.
48. Faedda GL, Tondo L, Baldessarini RJ, Suppes T, Tohen M: **Outcome after rapid vs gradual discontinuation of lithium treatment in bipolar disorders**. *Arch Gen Psychiatry* 1993, **50**(6):448-455.
49. Goodwin GM: **Evidence-based guidelines for treating bipolar disorder: revised second edition--recommendations from the British Association for Psychopharmacology**. *J Psychopharmacol (Oxf)* 2009, **23**(4):346-388.
50. Meltzer HY, Alphs L, Green AI, Altamura AC, Anand R, Bertoldi A, Bourgeois M, Chouinard G, Islam MZ, Kane J *et al*: **Clozapine treatment for suicidality in schizophrenia: International Suicide Prevention Trial (InterSePT)**. *Arch Gen Psychiatry* 2003, **60**(1):82-91.
51. Oquendo MA, Stanley B, Ellis SP, Mann JJ: **Protection of human subjects in intervention research for suicidal behavior**. *Am J Psychiatry* 2004, **161**(9):1558-1563.
52. Brookhart MA, Rassen JA, Wang PS, Dormuth C, Mogun H, Schneeweiss S: **Evaluating the validity of an instrumental variable study of neuroleptics: can between-physician differences in prescribing patterns be used to estimate treatment effects?** *Med Care* 2007, **45**(10 Supl 2):S116-122.
53. Stukel TA, Fisher ES, Wennberg DE, Alter DA, Gottlieb DJ, Vermeulen MJ: **Analysis of observational studies in the presence of treatment selection bias: effects of invasive cardiac management on AMI survival using propensity score and instrumental variable methods**. *JAMA* 2007, **297**(3):278-285.
54. Eng PM, Seeger JD, Loughlin J, Clifford CR, Mentor S, Walker AM: **Supplementary data collection with case-cohort analysis to address potential confounding in a cohort study of thromboembolism in oral contraceptive initiators matched on claims-based propensity scores**. *Pharmacoepidemiol Drug Saf* 2008, **17**(3):297-305.
